# Supplementary figures and images for: Alzheimer’s-Associated Upregulation of Mitochondria-Associated ER Membranes After Traumatic Brain Injury
Source: Cell Mol Neurobiol. 2022 Dec 26;43(5):2219–41. doi: 10.1007/s10571-022-01299-0 (PMC10287820; doi:10.1007/s10571-022-01299-0)

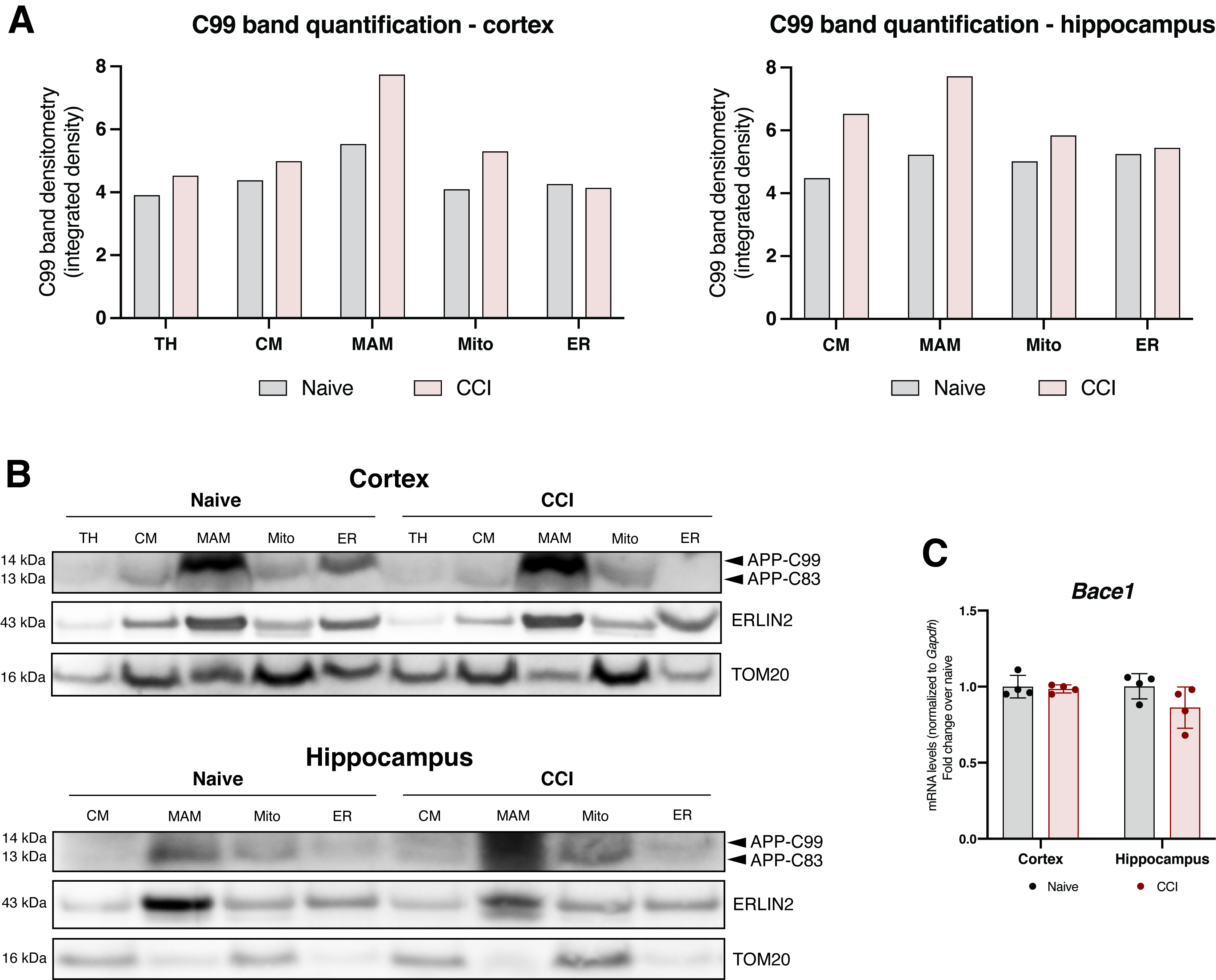

Supplement: Supplementary file 1 — Supplementary file1 (TIF 34813 KB) APP processing after CCI. (A) Quantification of C99 band intensity in both the cortex and hippocampus. (B) Subcellular fractionation of ipsilateral cortical and hippocampal homogenates 3d after CCI. TH, total homogenate; CM, crude mitochondria fraction; MAM, mitochondria-associated ER membranes fractions; Mito, purified mitochondria fraction; ER, bulk ER fraction. ERLIN-2 and TOM20 serve as MAM and mitochondria markers, respectively. This western blot is representative of 3 biological replicates, each conducted with pooled tissues from 4 mice/group. (C) Gene expression analysis via qPCR of Bace1, which encodes β-secretase (the enzyme responsible for C99 production from full-length APP), 3d after CCI, normalized to Gapdh. Each data point represents a separate mouse (biological replicate) and the average of 3 technical replicates (qPCR wells). Error bars represent standard deviation among biological replicates. Statistical comparisons are made to naïve (uninjured) tissues from the same region harvested and assayed alongside CCI tissues (two-tailed t-test, ⍺=0.05) [file 10571_2022_1299_MOESM1_ESM.tif]

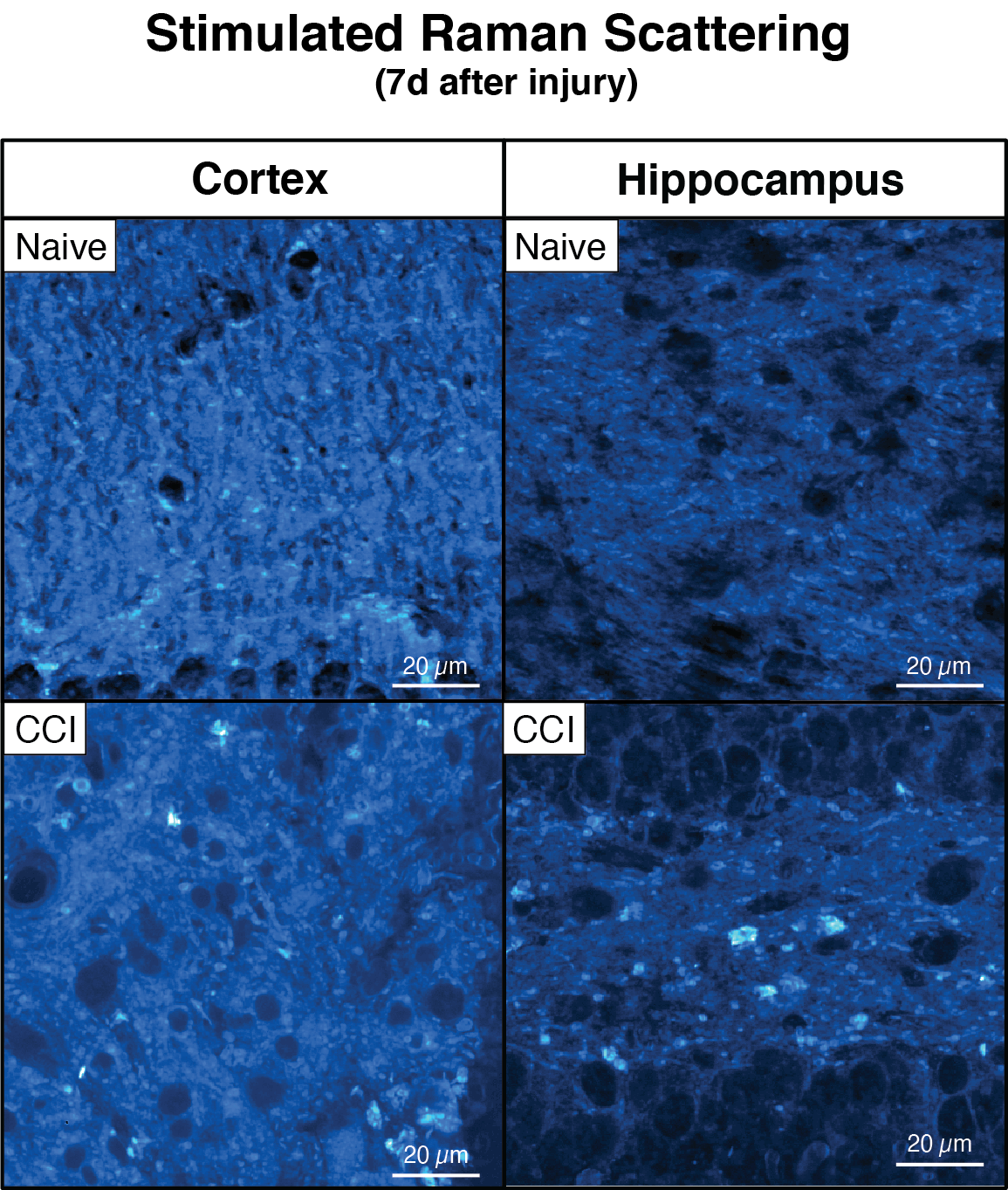

Supplement: Supplementary file 2 — Supplementary file2 (PDF 6154 KB) Stimulated Raman Scattering (SRS) imaging of lipid-specific C-H bonds 7d after CCI. Stimulated Raman scattering (SRS) microscopy image of C-H bonds in the lipid channel 7d after CCI, representative of 3 separate mice (biological replicates) per group. Lipid clusters are visible as bright puncta [file 10571_2022_1299_MOESM2_ESM.tif]

**A**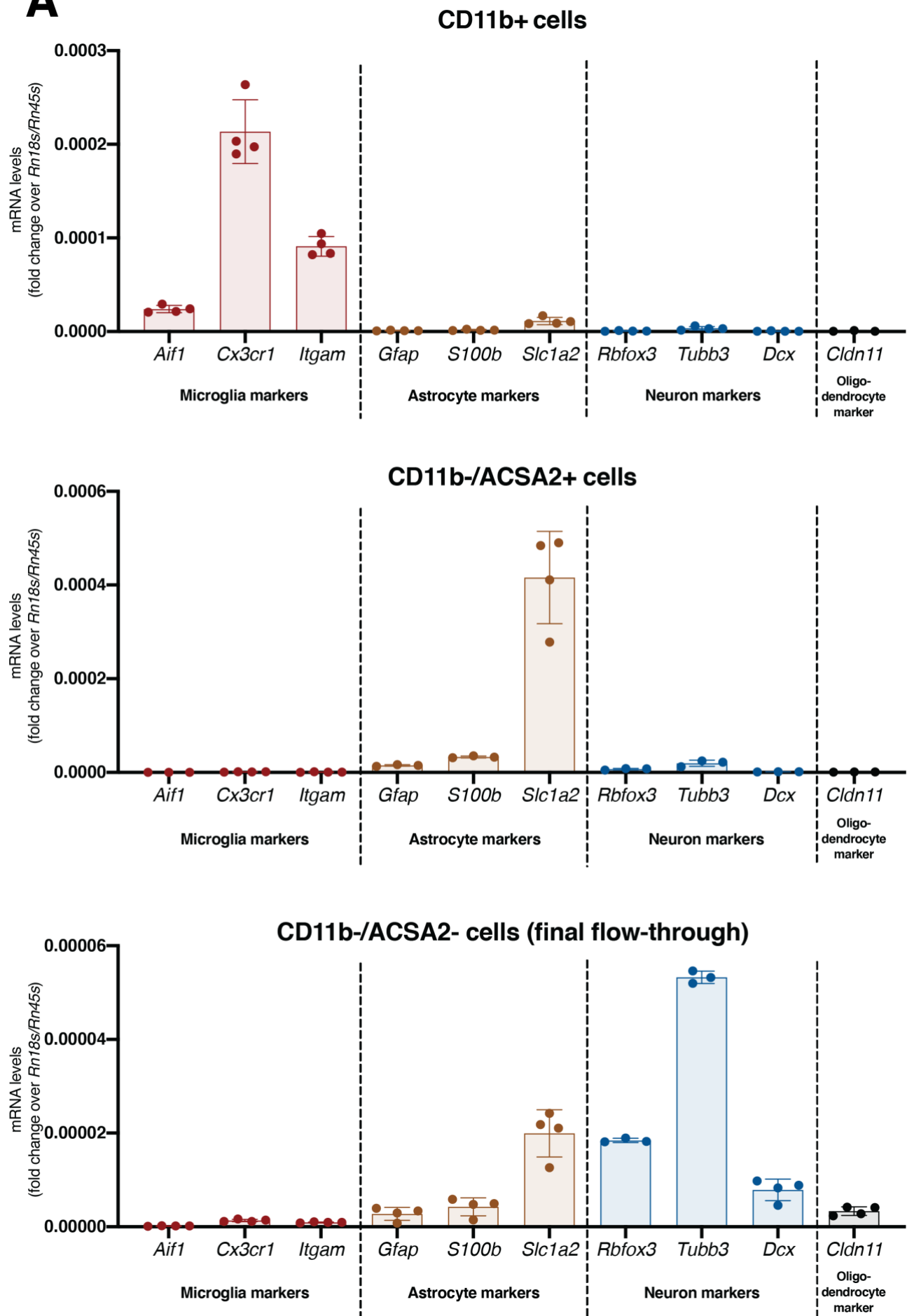

**B****Microglia markers:**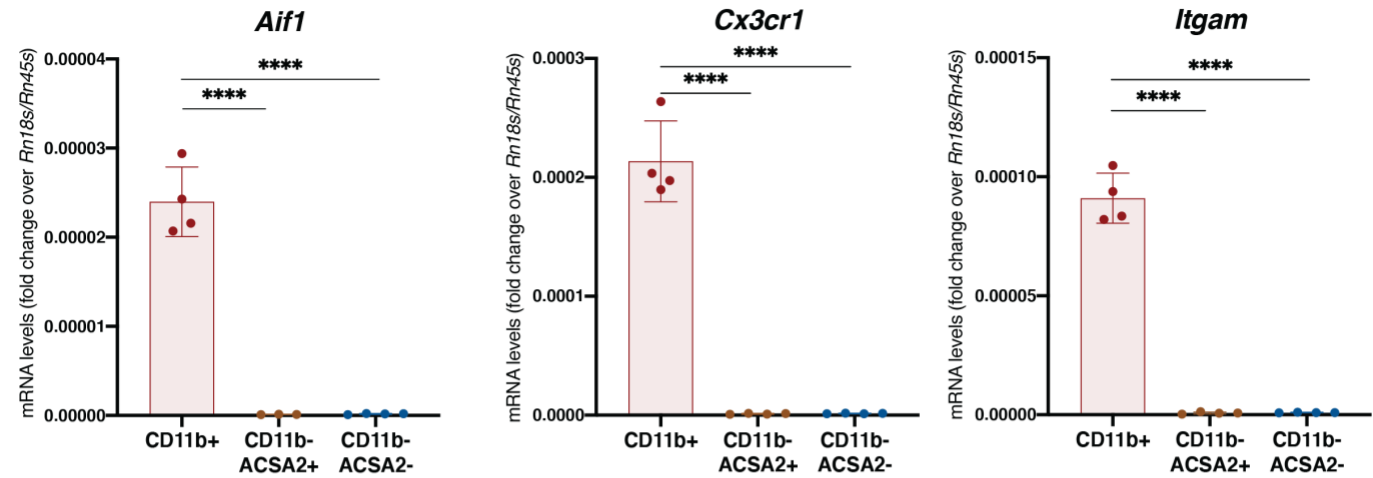**Astrocyte markers:**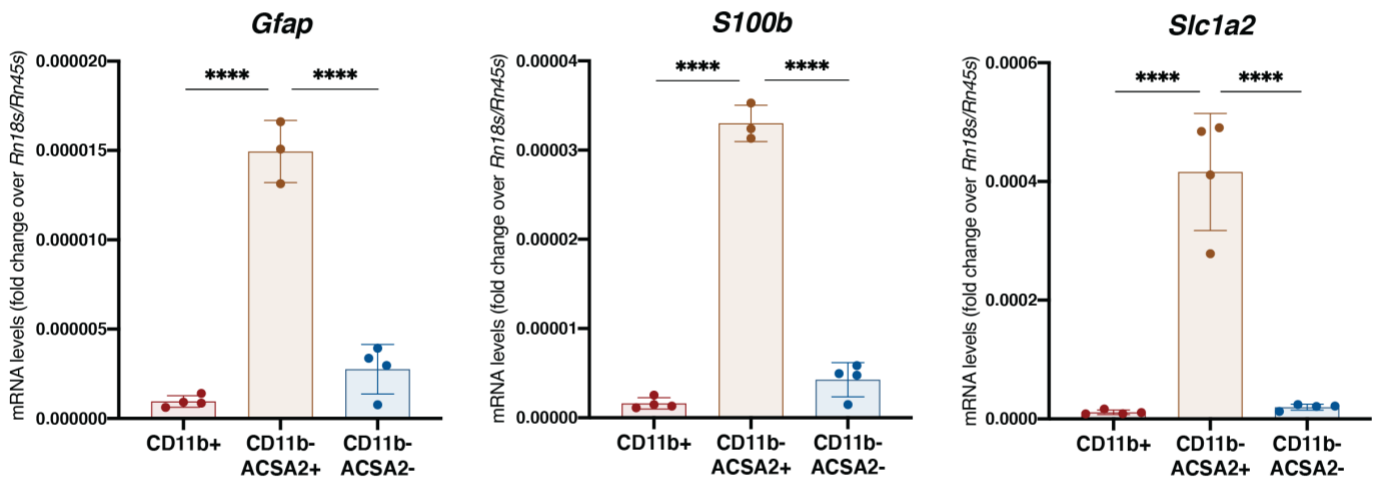**Neuron markers:**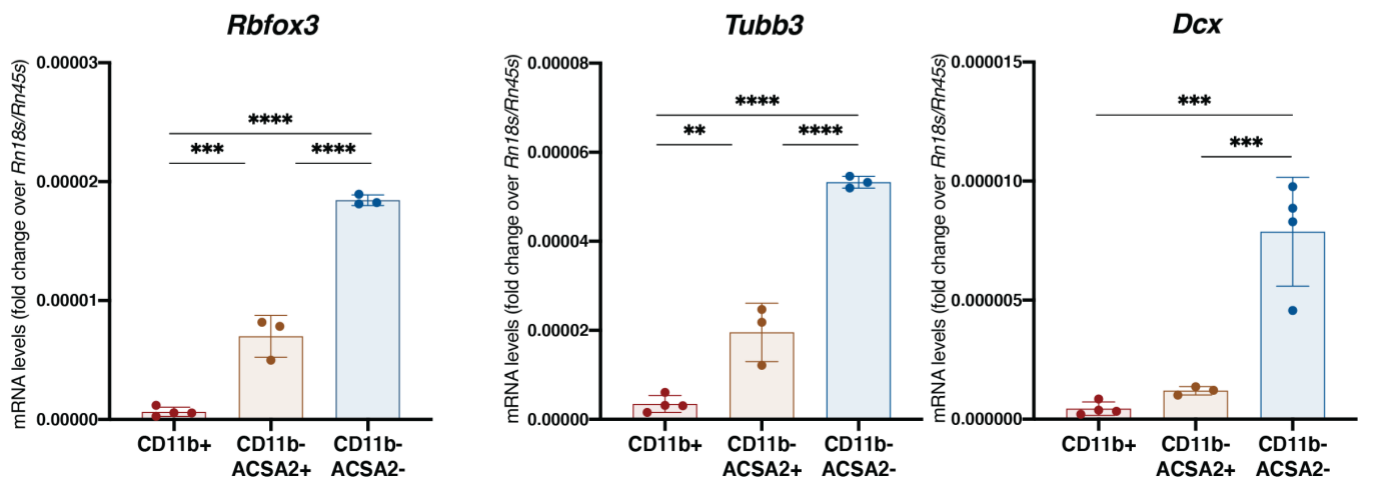

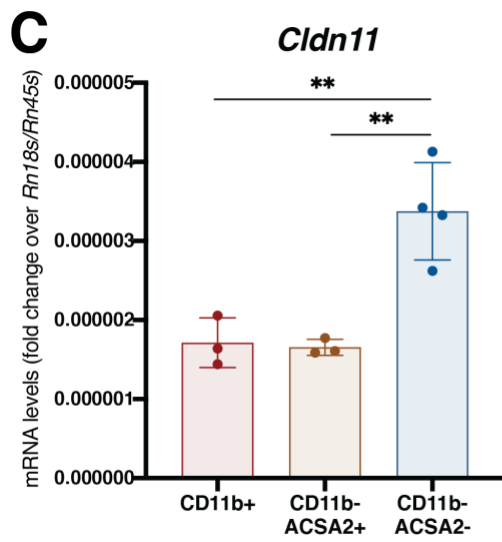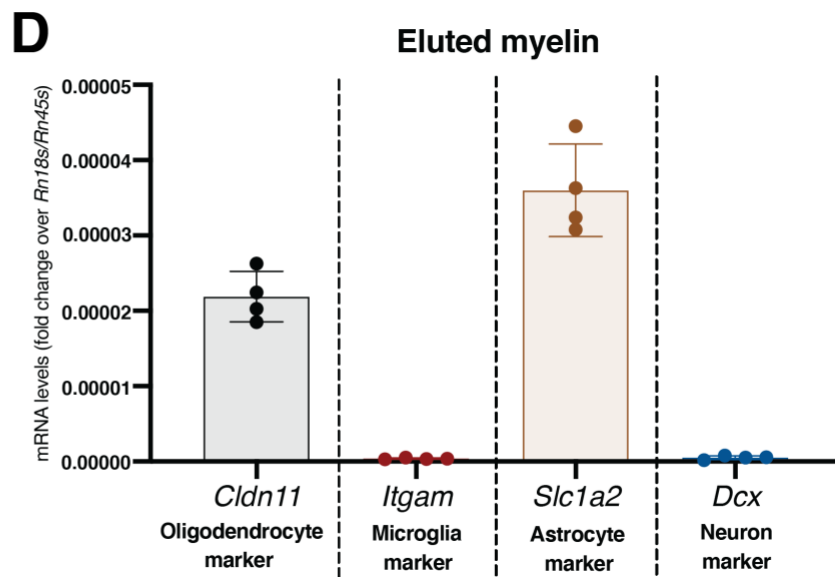

Supplement: Supplementary file 3 — Supplementary file3 (PDF 699 KB) Expression levels of cell type-specific mRNA markers in sorted cell populations from whole adult mouse brain. (A, B) Gene expression analysis of indicated genes in sorted ACSA-2+ (astrocytes) and CD11b+ (microglia) populations (collected after removal of myelin via magnetic beads) as well as remaining cells, considered to be enriched for neurons. The threshold-crossing cycle (Ct) value for each gene was normalized by the Ct value of Rn18s/Rn45s. The cell populations were each collected from 4 separate mice, with each data point representing the average of 3 technical replicates (qPCR wells) from each mouse. Error bars represent standard deviation among biological replicates. Statistical differences were determined by Ordinary one-way ANOVA followed by Bonferroni’s multiple comparisons test at an ⍺=0.05 significance level; *, p<0.5; **, p<0.01; ***, p<0.001; ****, p<0.0001. (A) Organized by population. (B) Organized by marker. (C, D) Cldn11 (protein: claudin-11, oligodendrocyte-specific protein, OSP) is used as an oligodendrocyte marker. (C) Gene expression analysis of Cldn11 in Cd11b+ (microglia), Cd11b-/ACSA-2+ (astrocyte) and Cd11b-/ACSA-2- (neuron) populations shows that final flow-through retains substantial Cldn11 expression compared to other populations. (D) Gene expression analysis of indicated cell type markers in eluted myelin shows that, while Cldn11 expression is higher than Itgam and Dcx, the myelin retains substantial expression of S100b (a gene known to also be enriched in oligodendrocytes) and Slc1a2 (an astrocyte marker) [file 10571_2022_1299_MOESM3_ESM.pdf]

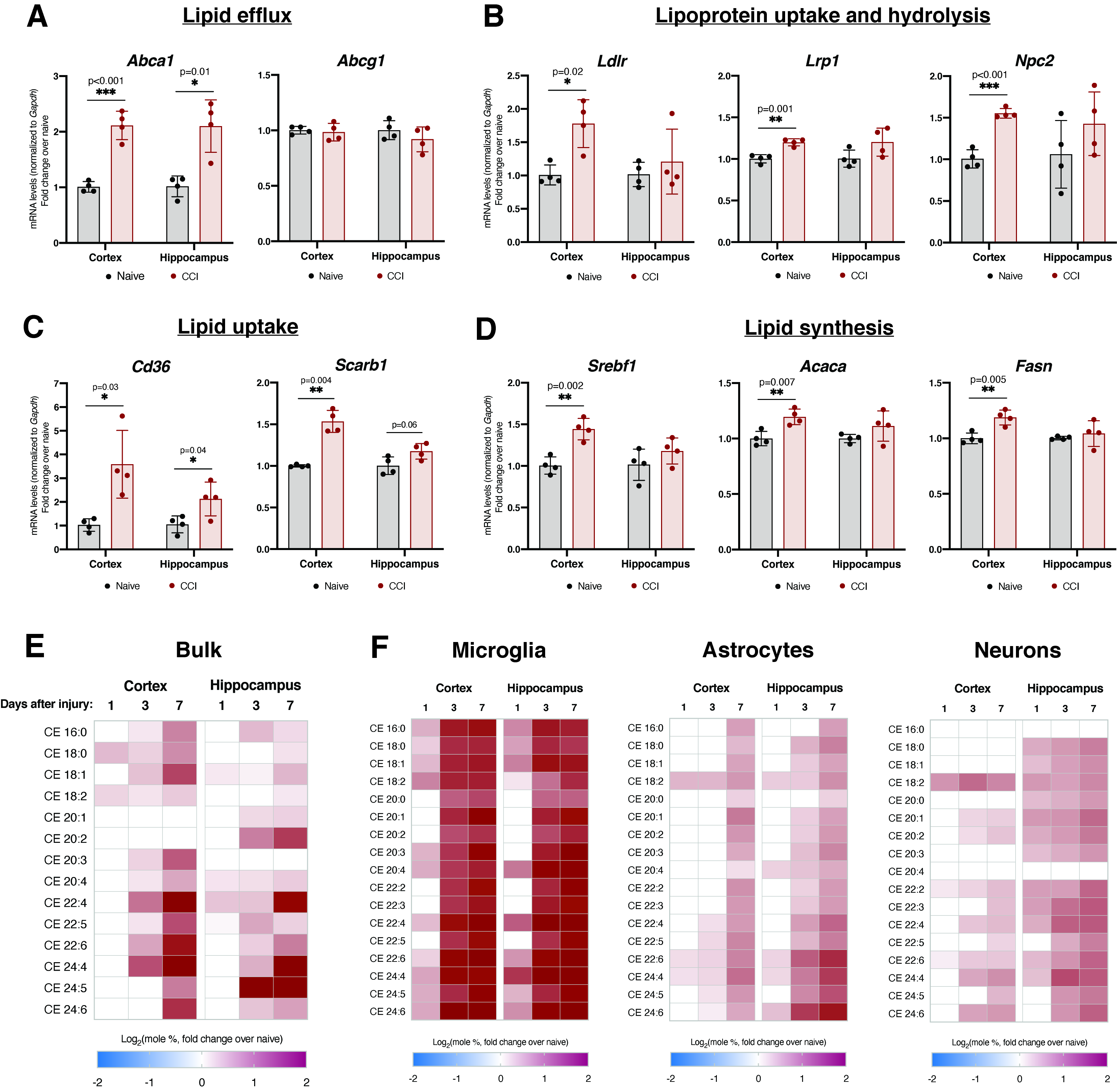

Supplement: Supplementary file 4 — Supplementary file4 (TIF 45438 KB) Increased lipid trafficking and cholesterol esterification in the cortex and hippocampus after CCI. (A-D) Expression of genes important in cellular lipid metabolism via qPCR 3d after CCI, normalized to Gapdh. (A) Lipid efflux transporters Abca1 and Abcg1. (B) Mediators of lipoprotein uptake, Ldlr and Lrp1, and hydrolysis, Npc2. (C) Scavenger lipid receptors Cd36 and Scarb1. (D) Regulators of de novo lipogenesis Srebf1, Acaca and Fasn. (E) Lipidomics levels of individual CE species in bulk ipsilateral cortical and hippocampal homogenates at multiple time-points after CCI. This data is averaged from 3 biological replicates. (F) Lipidomics levels of individual CE species in sorted populations of cortical and hippocampal microglia, astrocytes and neurons at multiple time-points after CCI. For A-F, statistical comparisons are made to naïve (uninjured) tissues from the same region and/or cell type harvested and assayed alongside CCI tissues (two-tailed t-test, ⍺=0.05, *, p<0.05; **, p<0.01). For A-D, each data point represents a separate mouse (biological replicate) and the average of 3 technical replicates (qPCR wells). Error bars represent standard deviation among biological replicates. For E and F, only statistically significant differences are represented (p<0.05) [file 10571_2022_1299_MOESM4_ESM.tif]

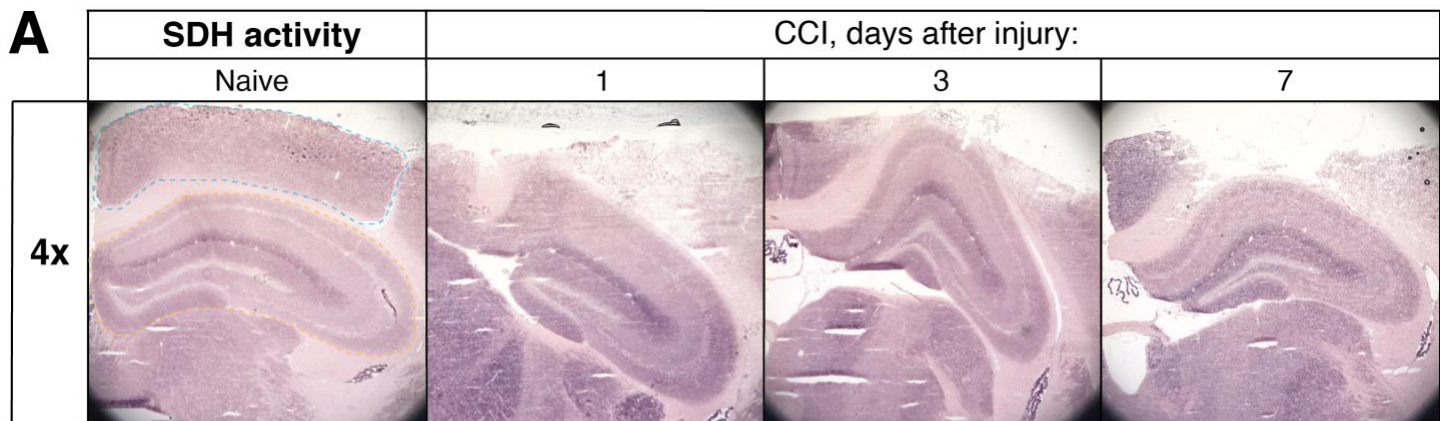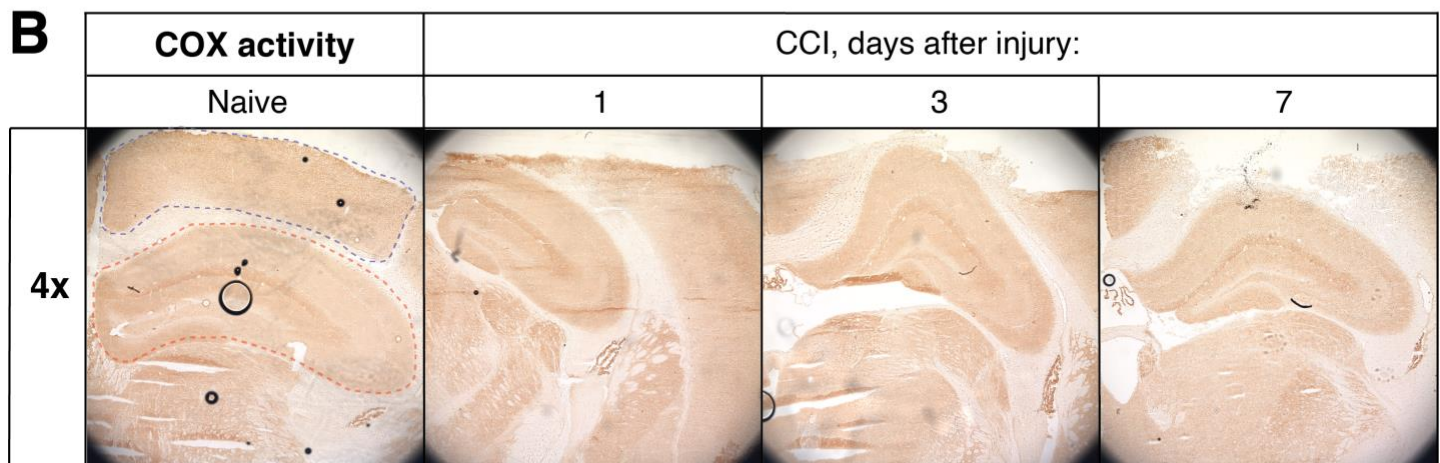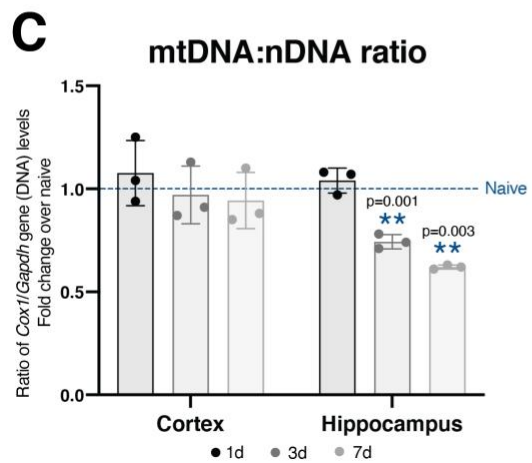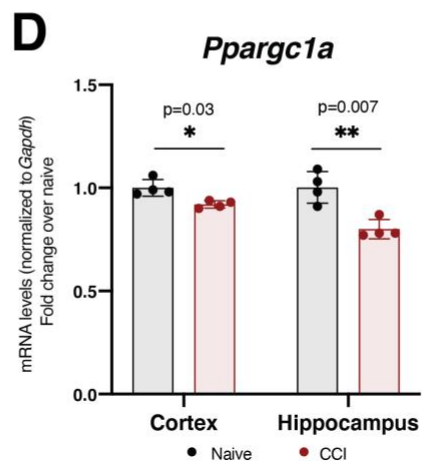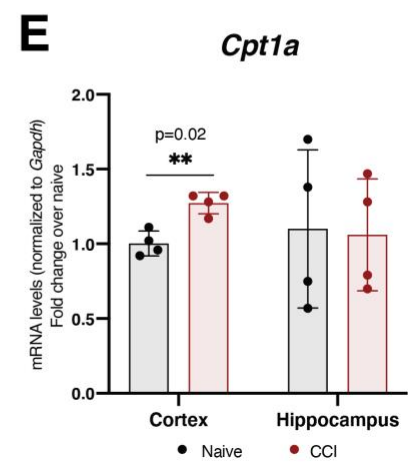

**F**

**Cortex**

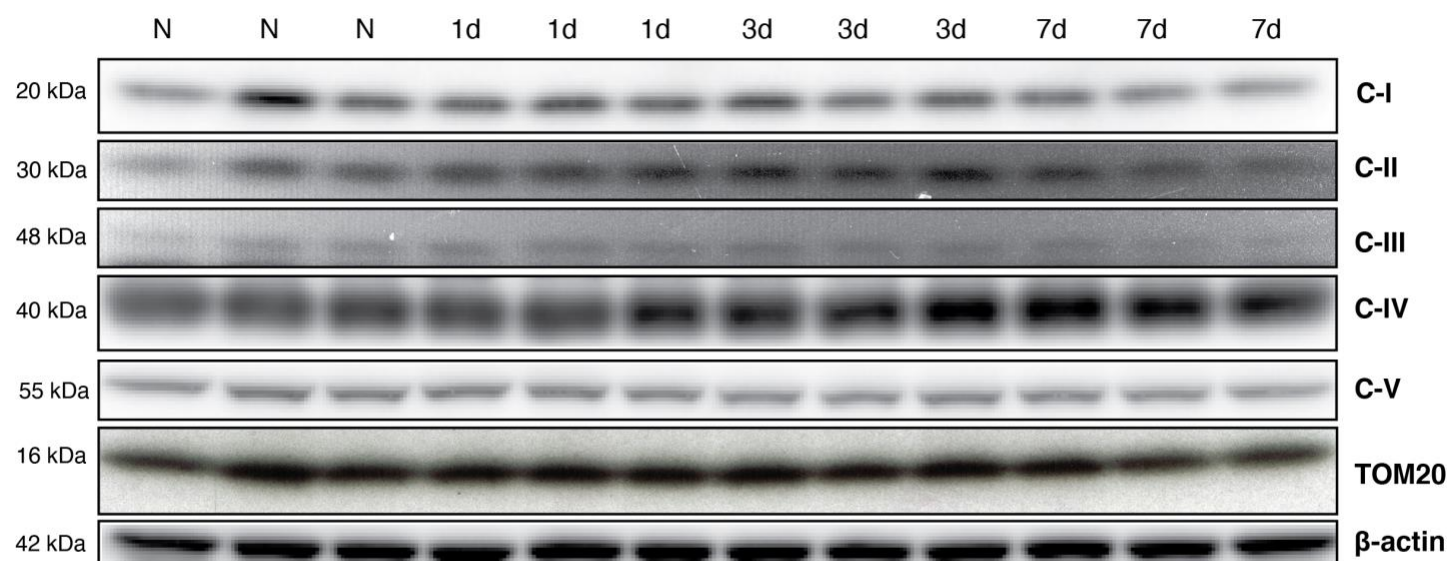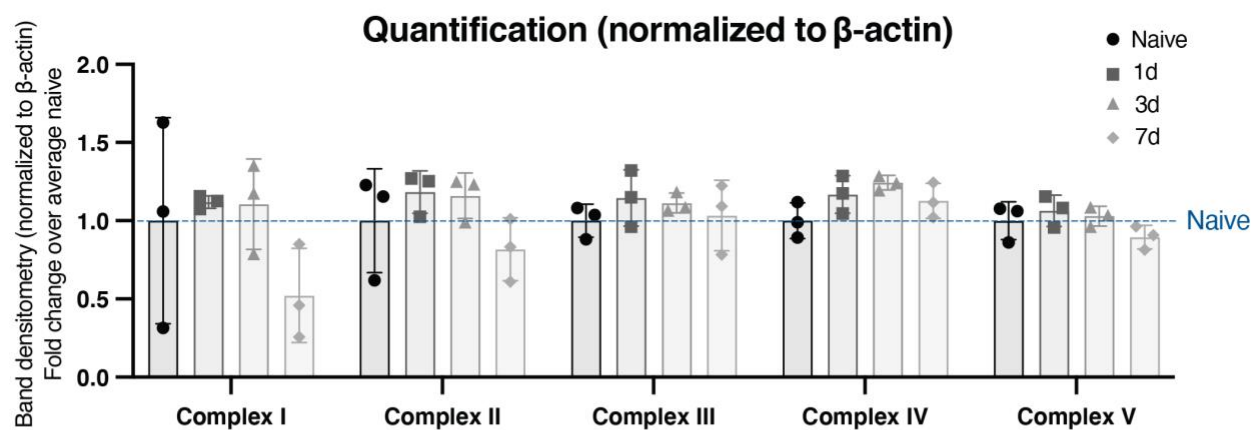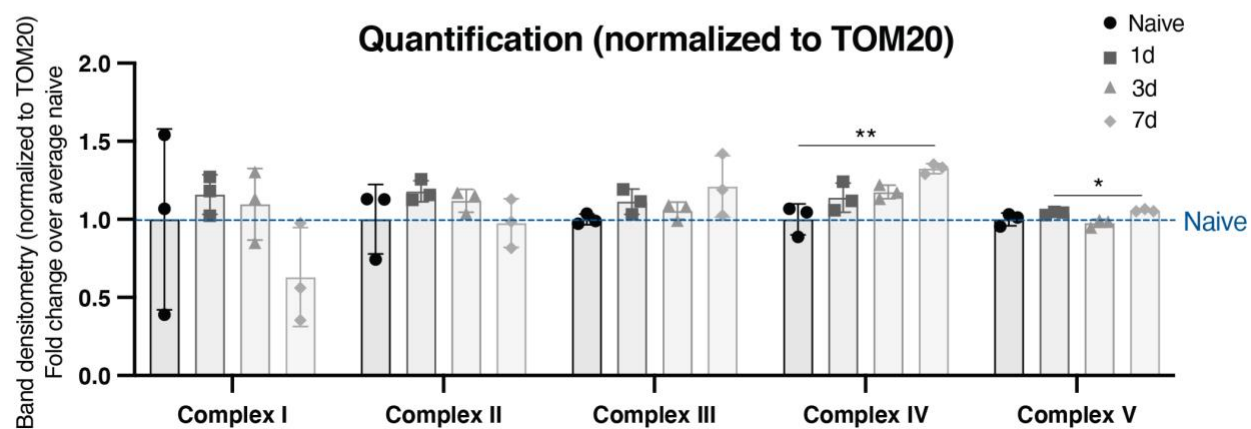

**G**

**Hippocampus**

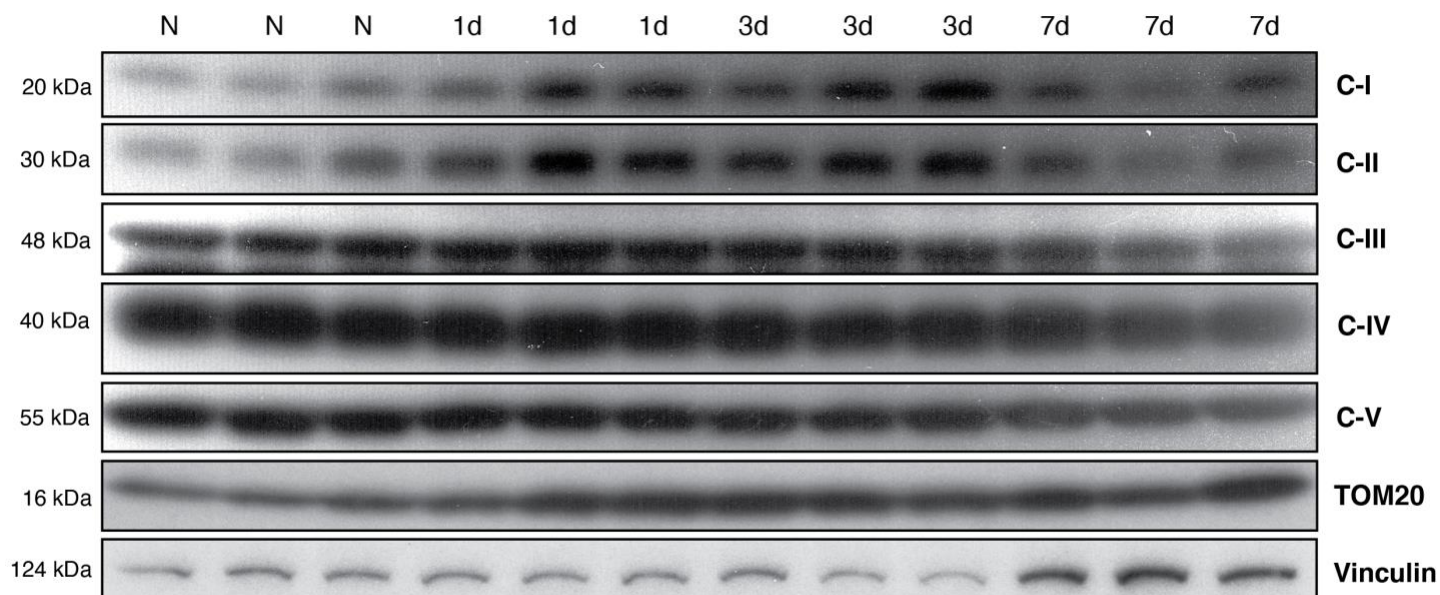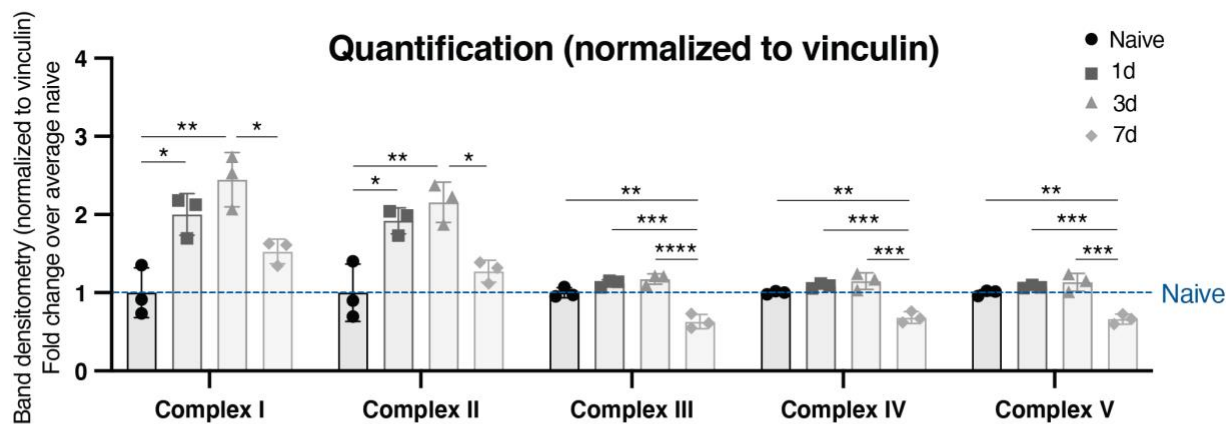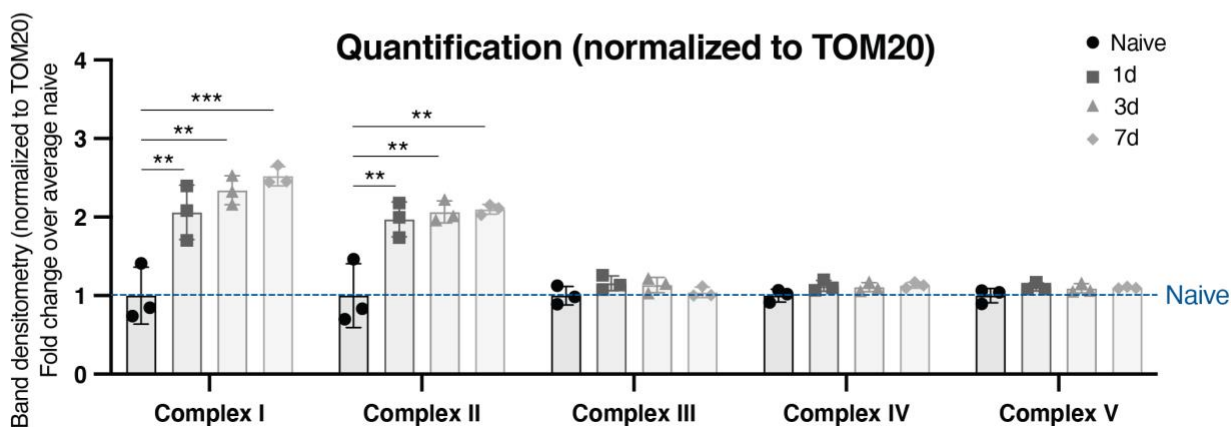

Supplement: Supplementary file 5 — Supplementary file5 (PDF 754 KB) Mitochondrial regulation after CCI. (A) Succinate dehydrogenase (SDH, complex-II) activity staining images in the ipsilateral cortex (outlined in blue) and ipsilateral hippocampus (outlined in yellow) at multiple time-points after CCI, representative of 3 separate mice (biological replicates) per group. (B) Cytochrome C oxidase (COX) activity staining images in the ipsilateral cortex (outlined in blue) and ipsilateral hippocampus (outlined in yellow) at multiple time-points after CCI, representative of 3 separate mice (biological replicates) per group. (C) Ratio of mitochondrial DNA (determined by Cox1 DNA quantity) to nuclear DNA (determined by Gapdh DNA quantity) in the ipsilateral cortex and hippocampus at multiple time-points after CCI. (D) Expression of Ppargc1a (encoding PGC1⍺), a regulator of mitochondrial biogenesis, via qPCR 3d after CCI. (E) Expression of Cpt1a, a regulator of mitochondrial fatty acid β-oxidation, via qPCR 3d after CCI. (F, G) Western blot of OxPhos complexes in both the cortex and hippocampus in naïve and CCI tissues (1, 3 and 7 days after CCI). Each lane is a separate mouse (biological replicate). Quantification is also included, with normalization to either β-actin or vinculin (whole cell markers) or TOM20 (mitochondria marker). For C-E, statistical comparisons are made to naïve (uninjured) tissues from the same region harvested and assayed alongside CCI tissues (two-tailed t-test; ⍺=0.05; *, p<0.05; **, p<0.01). Each data point represents a separate mouse (biological replicate) and the average of 3 technical replicates (qPCR wells). Error bars represent standard deviation among biological replicates. For F/G, statistical differences were determined by Ordinary one-way ANOVA followed by Bonferroni’s multiple comparisons test at an ⍺=0.05 significance level; *, p<0.5; **, p<0.01; ***, p<0.001. Error bars represent standard deviation among biological replicates [file 10571_2022_1299_MOESM5_ESM.pdf]

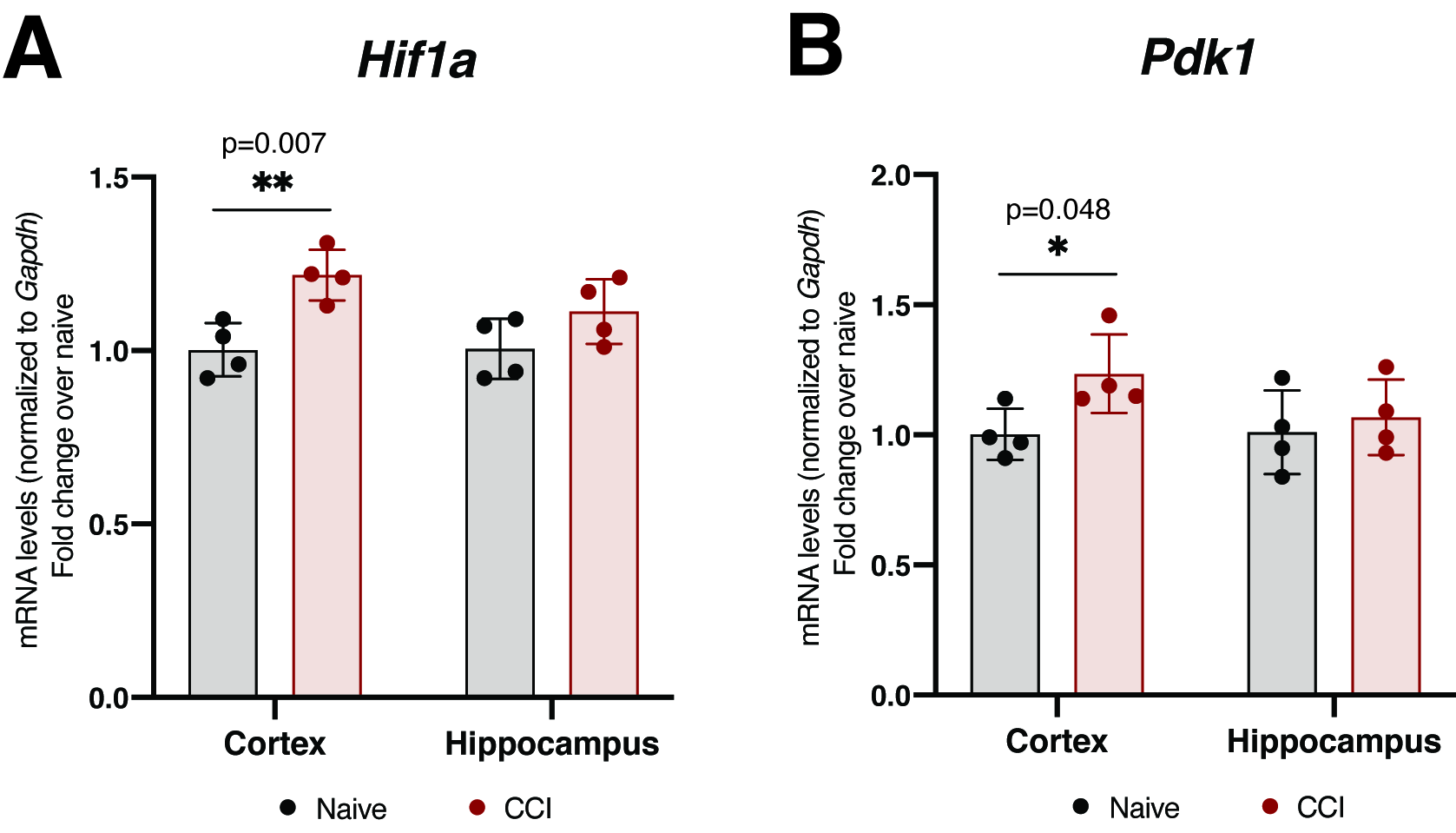

Supplement: Supplementary file 6 — Supplementary file6 (TIF 7370 KB) Expression of genes involved in hypoxic signaling and pyruvate regulation in the cortex and hippocampus after CCI. These qPCR analyses were conducted in ipsilateral cortical and hippocampal homogenates collected 3d after CCI. (A) Hif1a, a chief mediator of the hypoxic signaling pathway. (B) Pdk1, which encodes pyruvate dehydrogenase kinase 1. Statistical comparisons are made to naïve (uninjured) tissues from the same region harvested and assayed alongside CCI tissues (two-tailed t-test; ⍺=0.05; *, p<0.05; **, p<0.01). Each data point represents a separate mouse (biological replicate) and the average of 3 technical replicates (qPCR wells). Error bars represent standard deviation among biological replicates [file 10571_2022_1299_MOESM6_ESM.tif]
