# Supplementary material for: Alzheimer’s-Associated Upregulation of Mitochondria-Associated ER Membranes After Traumatic Brain Injury
Source: Cell Mol Neurobiol. 2022 Dec 26;43(5):2219–41. doi: 10.1007/s10571-022-01299-0 (PMC10287820; doi:10.1007/s10571-022-01299-0)
Supplement: Supplementary file 7 — Supplementary file7 (PDF 59 KB) Lipidomics source data - bulk [file 10571_2022_1299_MOESM7_ESM.pdf]

All values represent fold change over naïve samples

**Total of each lipid class:**	**Ipsilateral cortex**			**Ipsilateral hippocampus**		
**Days after injury:**	**1**	**3**	**7**	**1**	**3**	**7**
Free cholesterol (FC)	-0.3801534	-0.2111702	0.1009199	-0.1165221	-0.0540407	-0.5727573
Cholesteryl ester (CE)	0.97379671	1.06907205	2.84753999	0.87018658	1.37702307	-0.1674929
CE:FC	2.55611	1.75445	9.73742	1.98166	2.87817	0.16793
Sphingomyelin (SM)		0.20029526	0.00845426		0.25987045	0.63500771
Ceramide (Cer)		-0.9110107	-1.9279894		-1.4312742	-2
Monohydroxylated Cer (MHCer) + Ganglioside GM3	0.07040329	0.18659694	0.13653355	0.0659479	0.12486797	0.33047825
Monoglyceride (MG)	-0.1409022	-1.2132479	-2	-0.2501331	-0.1309348	-1.4571962
Diglyceride (DG)	0.2907	0.15259	-0.573	-0.22924	-0.3471	0.18873
Triglyceride (TG)	0.26186	0.35389	-0.1648	-0.68754	-0.2824	-0.08947
Phosphatidylcholine (PC)		0.28494439	0.04707204		0.09285902	0.16308906

**Cholesteryl esters (CEs):**	**Ipsilateral cortex**			**Ipsilateral hippocampus**		
**Days after injury:**	**1**	**3**	**7**	**1**	**3**	**7**
CE 16:0		0.64058831	2.5675983		1.60510653	0.92848555
CE 18:0	1.5772331	1.15378514	2.41442042			0.76518745
CE 18:1		1.45138316	3.51649413	0.58862831	0.40386778	1.66135447
CE 18:2	1.05241083	0.72097209	1.27838392			0.6118745
CE 20:1					0.96027464	1.12348535
CE 20:2					2.61849526	3.6531626
CE 20:3		1.09693978	3.19973016			
CE 20:4		0.67776103	1.95946106	0.69250999	0.84187207	1.24171604
CE 22:4		2.83135909	4.99699132	1.36475502	1.4098488	4.63080882
CE 22:5		0.71403871	3.39282245	0.25013024	1.97397223	1.14579851
CE 22:6		1.9886478	4.3753043		1.20184736	2.64891854
CE 24:4		3.35064695	5.77316072		1.81285055	4.93269545
CE 24:5			2.67111576		8.66654279	9.15296587
CE 24:6			3.85559898		1.38806623	2.07021375

**Acylcarnitines (ACs):**	**Ipsilateral cortex**			**Ipsilateral hippocampus**		
**Days after injury:**	**1**	**3**	**7**	**1**	**3**	**7**
AC C2:0	1.6580391	4.8360782	3.89844279		0.62769656	2.64441332
AC C3:0	0.8675618	2.89920911	2.4422593		0.91259936	3.01777261
AC C6:0		1.24336182	1.35818472	0.77152344	0.65179884	1.07935552
AC C12:0			1.93646988			
AC C14:0			1.83652217			
AC C16:0			3.08405693		0.74873488	1.49351121
AC C18:0						
AC C18:1		1.01139736	2.54930851		0.78190039	1.36295102

**Diacylglycerols (DGs):**	**Ipsilateral cortex**			**Ipsilateral hippocampus**		
**Days after injury:**	**1**	**3**	**7**	**1**	**3**	**7**
DG 30:0/14:0						
DG 30:1/14:0						
DG 32:0/16:0						
DG 32:1/16:0		1.09591213	1.10396008			
DG 32:2/16:1					0.51014549	0.97563145
DG 34:0/16:0						
DG 34:1/16:0						
DG 34:2/16:0		0.917404	1.10302385		0.97696106	0.08298899
DG 34:2/16:1						
DG 36:0/18:0						
DG 36:1/18:0		0.5286135	0.85356029	0.40842396	-0.2302126	-1.1645944
DG 36:2/18:0						-0.5121336
DG 36:2/18:1						-0.9280278
DG 36:3/18:1						-0.3634279
DG 36:4/18:0						0.92727969
DG 38:1/18:0		0.57277956	1.30753876	0.6164709	-0.6570594	-1.1148859
DG 38:2/18:0		0.49355671	1.41771995	0.21686686	0.84049646	1.10577875
DG 38:2/18:1		0.50731603	1.04492957	0.3088645	-0.4083384	-0.7959325
DG 38:3/18:0		0.75815236	0.8979186		0.39499789	1.24771764
DG 38:3/18:1						
DG 38:4/18:0			0.916892			
DG 38:4/18:1		1.13102664	1.04881262			
DG 40:4/18:0						
DG 40:4/18:1						
DG 40:5/18:0						
DG 40:5/18:1			1.17267433			-0.9633568
DG 40:6/18:0				-0.3239013	-1.2845674	-2.401739
DG 40:6/18:1						

Triacylglyerols (TGs):	Ipsilateral cortex			Ipsilateral hippocampus		
Days after injury:	1	3	7	1	3	7
TG 48:0/16:0						
TG 48:1/16:0						
TG 50:0/16:0						
TG 50:1/16:1						
TG 50:2/16:1						
TG 50:3/16:1					0.35775125	1.10055947
TG 52:0/18:0						
TG 52:1/18:0						
TG 52:2/18:0						
TG 52:3/18:1					0.26864348	0.93111873
TG 52:4/18:1			0.62450061			
TG 52:5/18:1			0.99682841	1.34528476	1.31283846	2.27633601
TG 52:5/20:4						
TG 54:0/18:0						
TG 54:1/18:0						
TG 54:2/18:0				-0.2841957	0.61890902	0.95758029
TG 54:3/18:0			0.94452972	-0.6378727	0.51389424	1.28280789
TG 54:4/18:1		0.47986003	0.43699278	-0.4133076	0.08368501	1.38791549
TG 54:4/20:4		0.29078126	0.70980231			
TG 54:5/18:1	0.36294474	0.58582115	0.65555745			0.92901956
TG 54:5/20:4	0.12391231	0.20421286	0.42813631			
TG 54:6/18:1					1.39563297	2.93472613
TG 54:6/20:4						
TG 54:7/18:1						1.67507775
TG 54:7/20:4	0.92686946	1.96355582	2.03495495			1.18504232
TG 56:3/18:1	0.45992744	0.79521832	1.28101612		0.25647864	0.90008933
TG 56:4/18:1						
TG 56:4/20:4						
TG 56:5/18:1				0.15764351	0.24676484	0.7462881
TG 56:5/20:4						
TG 56:6/20:4				0.08841455	0.23852648	-0.2245801
TG 56:7/20:4	1.21551795	1.29763885	0.92462438	0.04250137	0.29197159	-0.7721246
TG 56:8/20:4				0.11910416	0.55564913	-0.8713653
TG 56:9/20:4				1.60818792	0.98228877	-0.4615431
TG 58:5/20:4				1.35770441	1.16248782	1.35866422
TG 58:6/20:4						
TG 58:7/20:4			0.83456128			
TG 58:8/22:6	0.60205184	0.92326177	0.67946142			
TG 58:9/22:6						
TG 60:7/22:6						2.24821425
TG 60:8/22:6	0.81793391	1.07254244				2.19894177
TG 60:9/22:6	1.11873031	1.86802279	1.7371794			1.82897616
