# Supplementary material for: Alzheimer’s-Associated Upregulation of Mitochondria-Associated ER Membranes After Traumatic Brain Injury
Source: Cell Mol Neurobiol. 2022 Dec 26;43(5):2219–41. doi: 10.1007/s10571-022-01299-0 (PMC10287820; doi:10.1007/s10571-022-01299-0)
Supplement: Supplementary file 8 — Supplementary file8 (PDF 47 KB) Lipidomics source data - astrocytes [file 10571_2022_1299_MOESM8_ESM.pdf]

All values represent fold change over naïve samples						
**Total of each lipid class:**	**Ipsilateral cortex**			**Ipsilateral hippocampus**		
**Days after injury:**	**1**	**3**	**7**	**1**	**3**	**7**
Free cholesterol (FC)	-0.086196	-0.0187431	-0.3104425	-0.3163394	-0.4145157	-0.3745832
Cholesteryl ester (CE)	1.07277307	1.10431259	2.72204299	1.06689522	2.54630209	3.62000271
CE:FC	1.15896906	1.12305569	3.0324855	1.38323458	2.96081775	3.99458594
Sphingomyelin (SM)			1.59318762	1.03224506	0.94724311	0.62648455
Ceramide (Cer)		1.00107118	0.77991839	-0.3884012	-0.3318954	-0.433126
Monohydroxylated Cer (MHCer) + Ganglioside GM3	0.53372077	0.67157117	1.14207201	1.45578661	1.69457523	0.62628733
Monoglyceride (MG)	0.29894795	0.66045436	0.87722592	0.64254519	0.56965577	0.63453494
Diglyceride (DG)	0.30266526	-0.1886347	-0.4341915	1.07050377	1.33730222	0.98129559
Triglyceride (TG)	0.51436791	0.06274974	0.21120728	1.2374797	0.9839552	1.0151136
Phosphatidylcholine (PC)			1.58967692			1.25200794
**Cholesteryl esters (CEs):**	**Ipsilateral cortex**			**Ipsilateral hippocampus**		
**Days after injury:**	**1**	**3**	**7**	**1**	**3**	**7**
CE 16:0			0.84620885			0.851674
CE 18:0			0.64701167		0.70815826	1.0472267
CE 18:1			0.80981806		0.50413758	0.82336967
CE 18:2	0.67145006	0.66494216	0.89816816	0.49565169	0.51443597	0.95260351
CE 20:0			0.44921431			0.4477186
CE 20:1			1.08892979		0.48377381	0.84043324
CE 20:2			0.90822319		0.48627972	0.89717581
CE 20:3			0.7715964		0.6033482	1.01225882
CE 20:4			0.32695177	0.44659861	0.56742143	0.75098703
CE 22:2			0.94026748		0.44530145	0.87485509
CE 22:3			0.89254686		0.53541032	0.8761908
CE 22:4		0.33117232	0.8825332		0.85765227	1.20550343
CE 22:5		0.39112399	1.01029503		0.66840537	1.05873854
CE 22:6	0.3537833	0.43499952	1.02937421	0.42551674	1.26193761	1.56503583
CE 24:4	0.31654126	0.40929493	1.16137344	0.40133806	1.07189813	1.46073536
CE 24:5		0.30497456	0.9121443		0.99027681	1.33557249
CE 24:6		0.20429757	0.78020582	0.59217516	1.47200294	1.72416325
**Acylcarnitines (ACs):**	**Ipsilateral cortex**			**Ipsilateral hippocampus**		
**Days after injury:**	**1**	**3**	**7**	**1**	**3**	**7**
AC C2:0				1.42518629	2.18409884	
AC C3:0	1.47332768	1.19203039	0.49754329		1.73994166	
AC C6:0	0.77559369					
AC C12:0						
AC C14:0	1.13393385	0.84658417				
AC C16:0	1.33462669	0.95524928		1.85707109	1.78445322	1.84997909
AC C18:0	1.14200485			1.68859285	1.66975198	2.01634452
AC C18:1	1.02707637					

Diacylglycerols (DGs):	Ipsilateral cortex			Ipsilateral hippocampus		
**Days after injury:**	**1**	**3**	**7**	**1**	**3**	**7**
DG 34:1/16:0	0.2003429	0.60267463	0.98853604	1.46554642	1.43227213	2.05482766
DG 34:2/16:0	1.39433896	0.13699383	0.13007369	2.2705662	2.11066918	1.4774145
DG 36:1/18:0	0.71918352	0.02986352	-0.434983	1.62330718	1.77532489	1.39263499
DG 36:2/18:0	1.27460888	0.24767883	0.00531158	2.29082029	2.00341584	1.32124432
DG 36:2/18:1	0.35180771	-0.2205572	-0.4670022	1.20125041	1.39297382	1.15256811
DG 36:3/18:1	1.29858987	0.21784275	0.06130808	2.21311392	1.51425705	1.52342878
DG 38:2/18:1	0.6073977	-0.1606457	0.2105065	1.19614303	1.30834366	0.91716878
**Triacylglyerols (TGs):**	**Ipsilateral cortex**			**Ipsilateral hippocampus**		
**Days after injury:**	**1**	**3**	**7**	**1**	**3**	**7**
TG 52:3/18:1	1.92935916	1.41752651	1.92249547	2.26222638	1.45105978	1.28212004
TG 52:4/18:1	0.58294698	0.2116752	0.09706935	1.63628289	1.34314833	0.96280987
TG 52:5/18:1	0.9119555	0.35950121	0.16196219	1.97762476	1.49246673	0.98147057
TG 56:5/20:4	1.19805945	0.41324653	0.14595121	2.16082732	1.62497116	0.87477231
TG 56:6/20:4	0.69675112	0.30190058	-0.1410424	2.26635666	1.95380998	0.98648815
TG 56:7/20:4	0.93913114	0.55418014	-0.0627979	2.14142841	1.84849182	1.1163154
TG 56:8/20:4	0.64384239	0.17372582	0.39367823	1.7850795	1.68579979	1.2316925
TG 56:9/20:4	0.99737887	0.44781139	0.50964756	1.81106562	1.45098622	1.04094104
TG 58:5/20:4	0.82046328	0.34261876	0.11490649	1.77243206	1.50747522	0.87133585
TG 58:6/20:4	0.95304063	0.68557655	0.37626857	1.60989263	1.67582496	0.9991329
TG 58:7/20:4	0.93371288	0.60114733	0.1788039	1.99486588	1.98141327	1.29487476
TG 58:8/22:6	0.96375187	0.8324978	0.74935482	2.27001663	2.78415718	1.9109083
TG 58:9/22:6	1.17686336	0.79364347	1.0006918	1.79173929	1.7905914	1.18016887
TG 60:7/22:6	0.75594747	0.42535093	0.25344134	1.66407808	1.77296365	1.16133639
TG 60:8/22:6	1.3405557	0.62406538	0.19076067	2.22421616	1.79674771	1.10069145
TG 60:9/22:6	1.443239	0.73191805	0.56265362	1.9442133	1.47831189	0.9921153
