# Supplementary material for: Alzheimer’s-Associated Upregulation of Mitochondria-Associated ER Membranes After Traumatic Brain Injury
Source: Cell Mol Neurobiol. 2022 Dec 26;43(5):2219–41. doi: 10.1007/s10571-022-01299-0 (PMC10287820; doi:10.1007/s10571-022-01299-0)
Supplement: Supplementary file 9 — Supplementary file9 (PDF 49 KB) Lipidomics source data - microglia [file 10571_2022_1299_MOESM9_ESM.pdf]

All values represent fold change over naïve samples						
**Total of each lipid class:**	**Ipsilateral cortex**			**Ipsilateral hippocampus**		
**Days after injury:**	**1**	**3**	**7**	**1**	**3**	**7**
Free cholesterol (FC)	0.14882428	-0.3393934	-0.5596058	-0.5454888	-0.7832872	-1.1996666
Cholesteryl ester (CE)	2.37821436	5.52611352	6.13258771	3.29181441	5.94338866	6.37561505
CE:FC	2.22939008	5.86550689	6.6921935	3.8373032	6.72667583	7.57528165
Sphingomyelin (SM)		-0.2953701	-0.7239071	-0.1166636	-0.6858312	
Ceramide (Cer)		0.34859592	0.80394934	0.56231051	0.82444946	
Monohydroxylated Cer (MHCer) + Ganglioside GM3	0.51674634	0.26063861	-0.4208551	1.37141356	1.44645861	1.23732236
Monoglyceride (MG)	-0.0712384	-0.2917312	-0.86805		-0.5719425	-0.9919413
Diglyceride (DG)	0.79194689	0.83115712	0.05629368	0.49049908	-0.4313938	-0.8159418
Triglyceride (TG)	2.22272869	1.67885346	-0.1628497	1.53679084	0.51271121	-0.2900744
Phosphatidylcholine (PC)						
**Cholesteryl esters (CEs):**	**Ipsilateral cortex**			**Ipsilateral hippocampus**		
**Days after injury:**	**1**	**3**	**7**	**1**	**3**	**7**
CE 16:0	0.72741183	1.69983929	1.76703713	0.9986088	1.71661132	1.65104764
CE 18:0	0.48906551	1.56554078	1.6112384	0.8389311	1.59333822	1.49678947
CE 18:1	0.64731521	1.63261838	1.70310478	1.04141946	1.76507519	1.73679998
CE 18:2	1.02600775	1.62877892	1.64749514	0.27261587	1.14146296	1.5791201
CE 20:0		1.25501535	1.39471723		1.21524296	1.23131245
CE 20:1		1.6024512	1.92672695		1.64818047	1.80505781
CE 20:2		1.36012821	1.52579082		1.38557374	1.63290822
CE 20:3	0.69103041	1.50647431	1.88447602		1.67846044	2.01948569
CE 20:4	0.74144348	1.48008893	1.65335428	1.10371536	1.89704732	1.93983179
CE 22:2		1.54257244	1.80822252		1.59763161	1.91735424
CE 22:3		1.51826282	1.73456788		1.69297858	1.8213938
CE 22:4	0.62215888	1.78934559	1.96542462	1.26182883	2.29429717	2.33821274
CE 22:5		1.53877183	1.79673932		1.54121878	1.86300251
CE 22:6	0.45492299	1.86504761	2.07878627	0.91339066	1.86191621	2.12920916
CE 24:4	0.79577063	1.8666931	1.84767205	1.50076361	2.44127283	2.39631416
CE 24:5	0.74519034	1.56202379	1.89660679	0.69114342	1.59197287	1.89503889
CE 24:6	0.78112457	2.03072037	2.1661341	0.94922221	1.91688582	2.10502989
**Acylcarnitines (ACs):**	**Ipsilateral cortex**			**Ipsilateral hippocampus**		
**Days after injury:**	**1**	**3**	**7**	**1**	**3**	**7**
AC C2:0	1.55000774	2.99846189	1.06457635	0.68046245	1.81512291	1.52032452
AC C3:0						0.62874415
AC C6:0	0	0	0	0	0	0
AC C12:0						
AC C14:0	1.12691563	2.50656947	1.52473291			
AC C16:0	1.59564502	2.60502356	1.97038387	1.10360512	2.30796579	1.44464296
AC C18:0	2.01462447	3.77645348	2.40805167	1.50308326	2.80450239	1.6918076
AC C18:1	1.35879074	2.70898299	1.5457758			

**Diacylglycerols (DGs):**	**Ipsilateral cortex**			**Ipsilateral hippocampus**		
**Days after injury:**	**1**	**3**	**7**	**1**	**3**	**7**
DG 34:1/16:0			0.30572944	0.34244331	-0.802407	-1.1548327
DG 34:2/16:0	2.34091917	2.32348674	0.49567932	1.73803845	0.16869319	-0.4218023
DG 36:1/18:0	1.24274753	1.51213543	0.16808331	1.32631409	0.60581712	-0.4469639
DG 36:2/18:0	2.18842851	2.29692066	0.36327966	1.94181701	0.39579723	-0.4122433
DG 36:2/18:1	1.14257081	1.07521234	-0.2377663	0.39498977	-0.7195493	-1.6138015
DG 36:3/18:1	3.084278	3.2417028	0.90724216	1.17177465	-0.2487866	-0.9441931
DG 38:2/18:1	0.28384783	0.08667362	-1.0145405	0.1595029	-1.4817857	-1.2608
**Triacylglyerols (TGs):**	**Ipsilateral cortex**			**Ipsilateral hippocampus**		
**Days after injury:**	**1**	**3**	**7**	**1**	**3**	**7**
TG 52:3/18:1	1.38944952	1.17673918		1.12984278		0.29595884
TG 52:4/18:1	3.06202396	3.11551846		2.37800117	1.46396164	-0.684067
TG 52:5/18:1	3.57314967	3.01767105		3.14381103	1.31933279	-0.535074
TG 56:5/20:4	3.3203921	2.81826915		3.17544658	1.00140518	-0.3378561
TG 56:6/20:4	2.34949677	1.67083023		2.59966969	0.85327734	-0.5021159
TG 56:7/20:4	2.96986106	2.13497208		3.17522512	0.97668188	-0.536131
TG 56:8/20:4	2.39946947	3.34633936		1.6716423	1.21806198	-0.4551967
TG 56:9/20:4	2.91591829	3.35322001	0.59097671	2.31953521	1.37332087	-0.404448
TG 58:5/20:4	2.4657819	2.70157299	-0.0123327	2.2753614	1.10296921	-0.5293892
TG 58:6/20:4	4.02374992	4.34802696	0.20721576	3.04395652	2.3433031	-0.5083695
TG 58:7/20:4	3.17385096	3.72300176	0.02729351	2.99897563	1.84415383	-0.3703041
TG 58:8/22:6	3.30564242	5.20531501	2.55275781	2.14707519	2.51266125	0.32020035
TG 58:9/22:6	2.99593152	4.38375227	1.22546943	4.62155355	4.31632135	2.09977606
TG 60:7/22:6	2.81428591	3.75575605	0.39407169	2.33059568	1.76397993	-0.6156973
TG 60:8/22:6	2.94713863	2.37898357	-0.2358589	2.29772533	0.51710543	-0.8421006
TG 60:9/22:6	3.23374328	2.61303633	-0.1455517	2.79053528	0.64573794	-0.6386789

