# Supplementary material for: Alzheimer’s-Associated Upregulation of Mitochondria-Associated ER Membranes After Traumatic Brain Injury
Source: Cell Mol Neurobiol. 2022 Dec 26;43(5):2219–41. doi: 10.1007/s10571-022-01299-0 (PMC10287820; doi:10.1007/s10571-022-01299-0)
Supplement: Supplementary file 10 — Supplementary file10 (PDF 47 KB) Lipidomics source data - neurons [file 10571_2022_1299_MOESM10_ESM.pdf]

All values represent fold change over naïve samples						
**<u>Total of each lipid class:</u>**	**Ipsilateral cortex**			**Ipsilateral hippocampus**		
**Days after injury:**	**1**	**3**	**7**	**1**	**3**	**7**
Free cholesterol (FC)	-0.0474476	0.03493972	-0.0956349	-0.9693679	-0.9336163	-0.663681
Cholesteryl ester (CE)	0.44029786	1.15977684	1.17411	1.95023011	2.41059532	3.02959146
CE:FC	0.48774544	1.12483712	1.26974494	2.91959805	3.34421165	3.69327247
Sphingomyelin (SM)		0.86757438	1.66149552	1.5301011	1.48242568	1.22010011
Ceramide (Cer)		0.95423849	0.84922179	-1.7646748	-1.7468525	-1.3109063
Monohydroxylated Cer (MHCer) + Ganglioside GM3	-0.0960119	-0.4016752	-0.5138069	1.58421169	1.62036136	0.73982214
Monoglyceride (MG)	-0.0105085	-0.353371	0.86457882	0.94728445	1.11237797	1.42916884
Diglyceride (DG)	-0.2861985	-0.1000616	-0.6872579	0.03742771	-0.1140908	-0.1434709
Triglyceride (TG)	-0.1199492	-0.201325	-0.1968648	0.68874447	0.94254442	0.14437927
Phosphatidylcholine (PC)		0.81702399	1.6209849		3.07162762	2.61006903
**<u>Cholesteryl esters (CEs):</u>**	**Ipsilateral cortex**			**Ipsilateral hippocampus**		
**Days after injury:**	**1**	**3**	**7**	**1**	**3**	**7**
CE 16:0						
CE 18:0				0.75881629	0.8892578	1.05003012
CE 18:1				0.61067952	0.83655048	0.95981708
CE 18:2	0.91978839	1.17721873	1.00157912	0.8945703	0.83287103	0.99082069
CE 20:0				0.6268618	0.72855395	0.89043997
CE 20:1		0.4159602	0.48024051	0.89194381	0.92143612	1.18334193
CE 20:2		0.358515	0.56307661	0.75842998	0.87622638	1.09323138
CE 20:3				0.61099416	0.73318058	0.91003941
CE 20:4						
CE 22:2	0.24748835	0.42932732	0.61815364	0.85607252	0.85401146	1.21031677
CE 22:3			0.59947948	0.85572513	1.15090558	1.28482414
CE 22:4		0.54755734	0.636468	0.73914093	1.24149972	1.27576327
CE 22:5			0.42772617		0.75777629	1.05255505
CE 22:6	0.18971624	0.52290524	0.53876604	0.63910648	0.92276254	1.22124284
CE 24:4		0.78877595	0.94626541	0.58844241	1.36424152	1.27935247
CE 24:5			0.66419675		0.91363349	1.09388683
CE 24:6		0.81913351	0.89739995		0.91851936	1.23344799
**<u>Acylcarnitines (ACs):</u>**	**Ipsilateral cortex**			**Ipsilateral hippocampus**		
**Days after injury:**	**1**	**3**	**7**	**1**	**3**	**7**
AC C2:0			1.40430846		0.70700889	1.67642118
AC C3:0	0.88877829	1.09561686	1.20967309			1.71340506
AC C6:0	1.09516545	1.29174059	1.59740678	0.92483645	1.53396974	2.47444065
AC C12:0						
AC C14:0		1.31747871			0.7200373	
AC C16:0	1.07913015	1.53707637			1.93300759	
AC C18:0	0.90406098	1.49143797			1.91885807	1.25346136
AC C18:1	0.95947162	1.26567008			1.25224413	

Diacylglycerols (DGs):	Ipsilateral cortex			Ipsilateral hippocampus		
**Days after injury:**	**1**	**3**	**7**	**1**	**3**	**7**
DG 34:1/16:0	0.00812566	-0.1425053	-0.1439297	1.09439534	0.88237304	1.32223296
DG 34:2/16:0	0.50713317	0.29890753	-0.1581742	1.82794085	1.25235601	1.06462897
DG 36:1/18:0	-0.0347053	0.27513336	-0.7168712	1.04298159	0.62562672	0.41253359
DG 36:2/18:0	0.561848	0.33569167	-0.0235304	2.03295572	1.38232882	1.07411462
DG 36:2/18:1	-0.118639	0.05150681	-0.3994335	0.70360068	0.57322713	0.39277698
DG 36:3/18:1	0.61651363	0.11356977	-0.0427577	1.87730204	1.22379841	1.00195695
DG 38:2/18:1	0.09550208	-0.0449832	-0.137919	1.74432226	1.34602238	1.80190843
**Triacylglyerols (TGs):**	**Ipsilateral cortex**			**Ipsilateral hippocampus**		
**Days after injury:**	**1**	**3**	**7**	**1**	**3**	**7**
TG 52:3/18:1	1.73674837	1.35643267	1.29399409	3.05820886	3.60253618	3.19714445
TG 52:4/18:1	-0.0674054	-0.1291138	-0.2538524	1.32102483	1.44193799	0.36302184
TG 52:5/18:1	0.19431032	0.19656493	0.25545047	1.91578521	2.0487853	0.97773608
TG 56:5/20:4	0.21201438	0.08050535	0.21959166	1.71956933	1.99611798	0.76952527
TG 56:6/20:4	-0.1733944	0.08822731	-0.0902261	1.57108827	2.04357373	0.72301924
TG 56:7/20:4	0.05774702	0.2693862	0.3694244	1.68724411	1.98920106	0.58196543
TG 56:8/20:4	0.34058091	-0.0509507	0.00078129	1.9586548	1.96995779	0.77437048
TG 56:9/20:4	0.27936082	0.06539186	0.10141681	2.21522987	2.03610416	1.2017785
TG 58:5/20:4	0.13310952	0.05314384	0.20271654	1.71727619	1.94706526	0.91868626
TG 58:6/20:4	0.23997068	0.33787932	0.05546828	1.91149267	2.02080016	0.71297127
TG 58:7/20:4	0.16191285	0.25488739	0.04363859	1.57733068	1.74174102	0.63358797
TG 58:8/22:6	0.2888732	0.42021954	-0.0758481	1.87710849	2.11663576	0.77723568
TG 58:9/22:6	0.37445317	0.33034939	0.12358472	2.16704019	2.05876892	0.95047647
TG 60:7/22:6	0.08850003	0.02547042	-0.0403956	1.54448337	1.70626719	0.53919557
TG 60:8/22:6	0.44841176	0.44624632	0.36396066	1.77554599	2.12270567	1.01335374
TG 60:9/22:6	0.33430853	0.21464164	0.17353032	1.96377516	2.0645275	1.07419632
