# Supplementary material for: Alzheimer’s-Associated Upregulation of Mitochondria-Associated ER Membranes After Traumatic Brain Injury
Source: Cell Mol Neurobiol. 2022 Dec 26;43(5):2219–41. doi: 10.1007/s10571-022-01299-0 (PMC10287820; doi:10.1007/s10571-022-01299-0)

Fig. 2A: Western blot for APP-C99 (cortex)

M3.2 (Biolegend 805701) to detect APP-C99 (14 kDa)

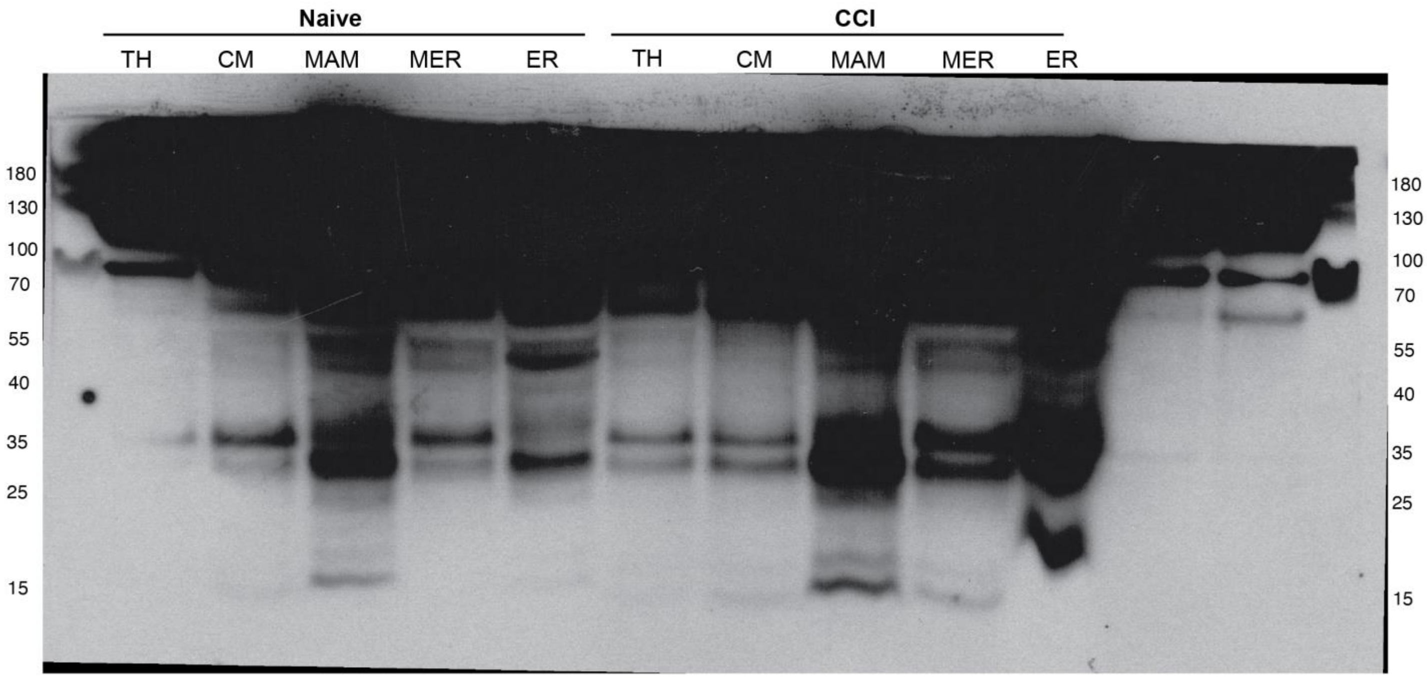

APP C-terminus (Sigma A8717) to detect APP-C99 (14 kDa) and APP-C83 (13 kDa)

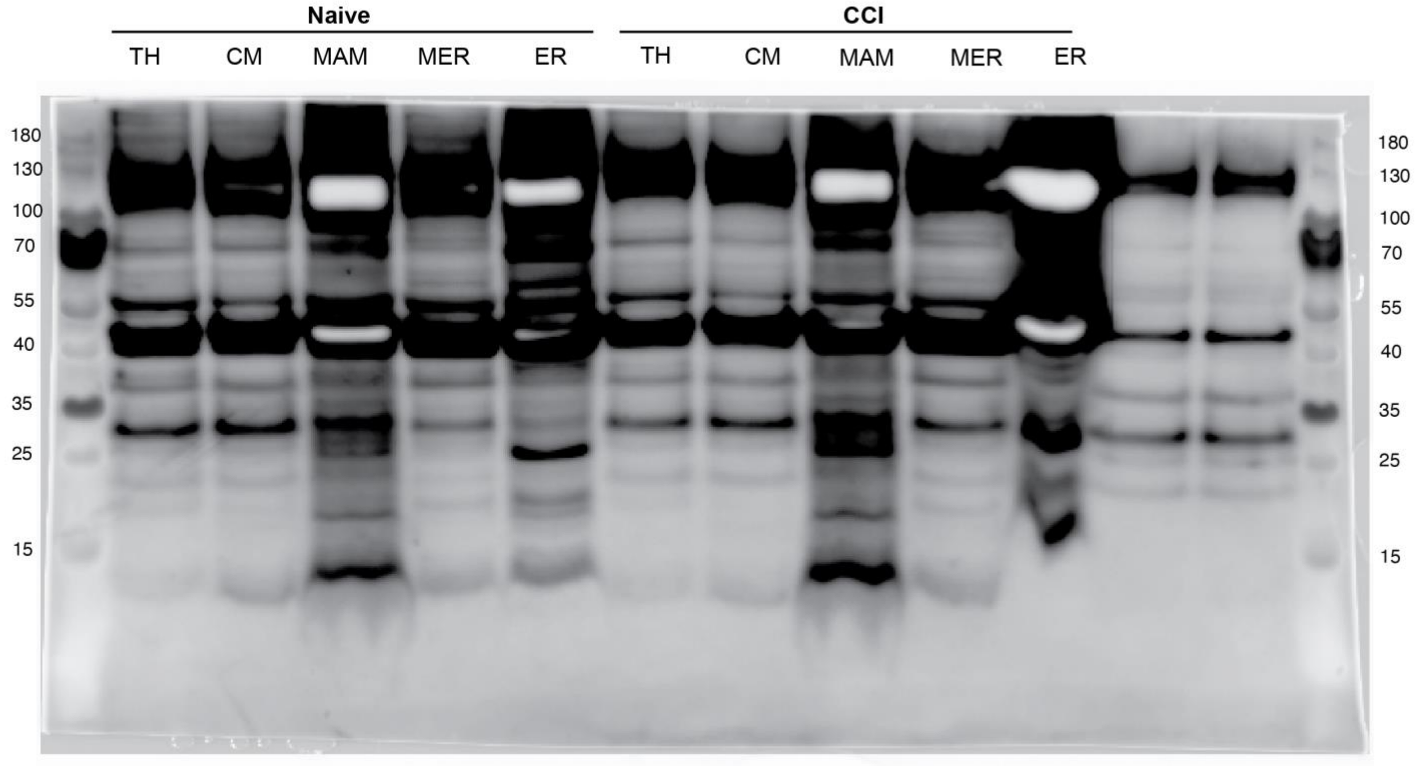

Erlin-2 (Abcam Ab129207) - 43 kDa

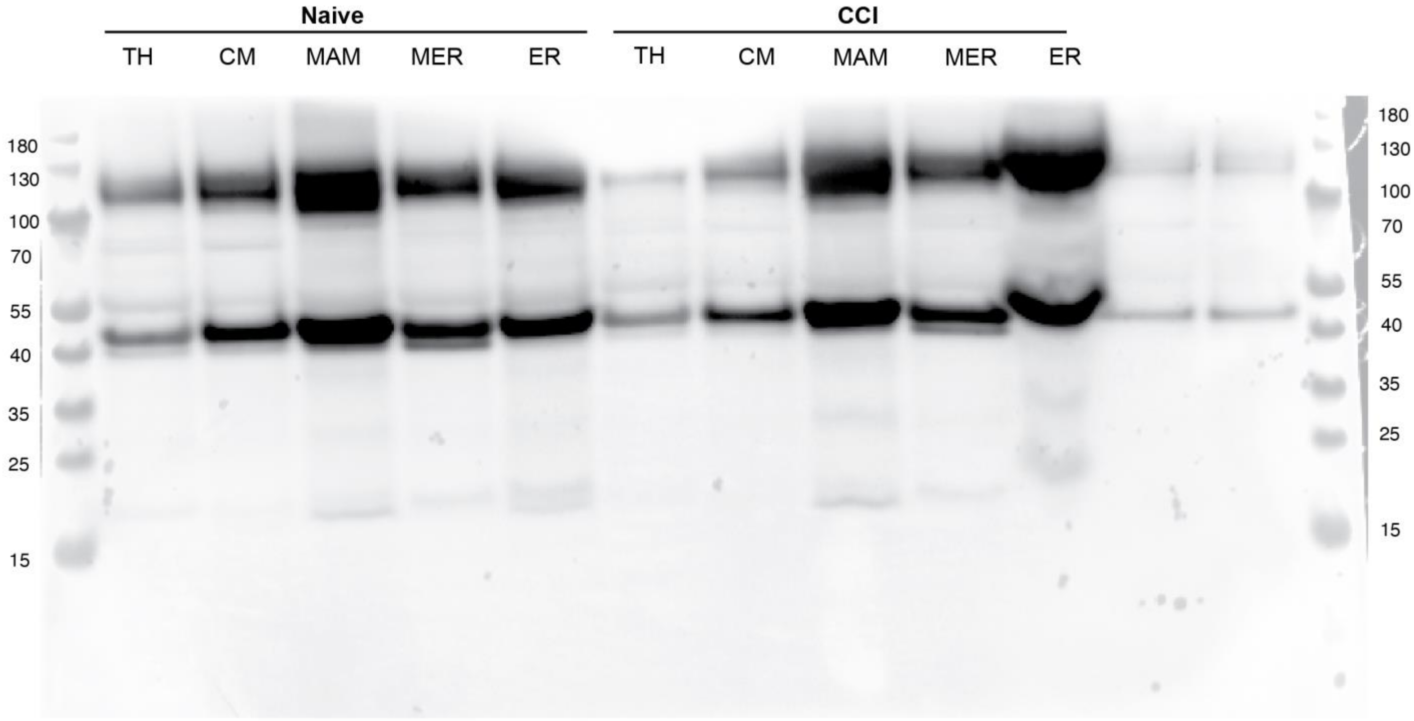

TOM20 (Santa Cruz sc-11415) - 16 kDa

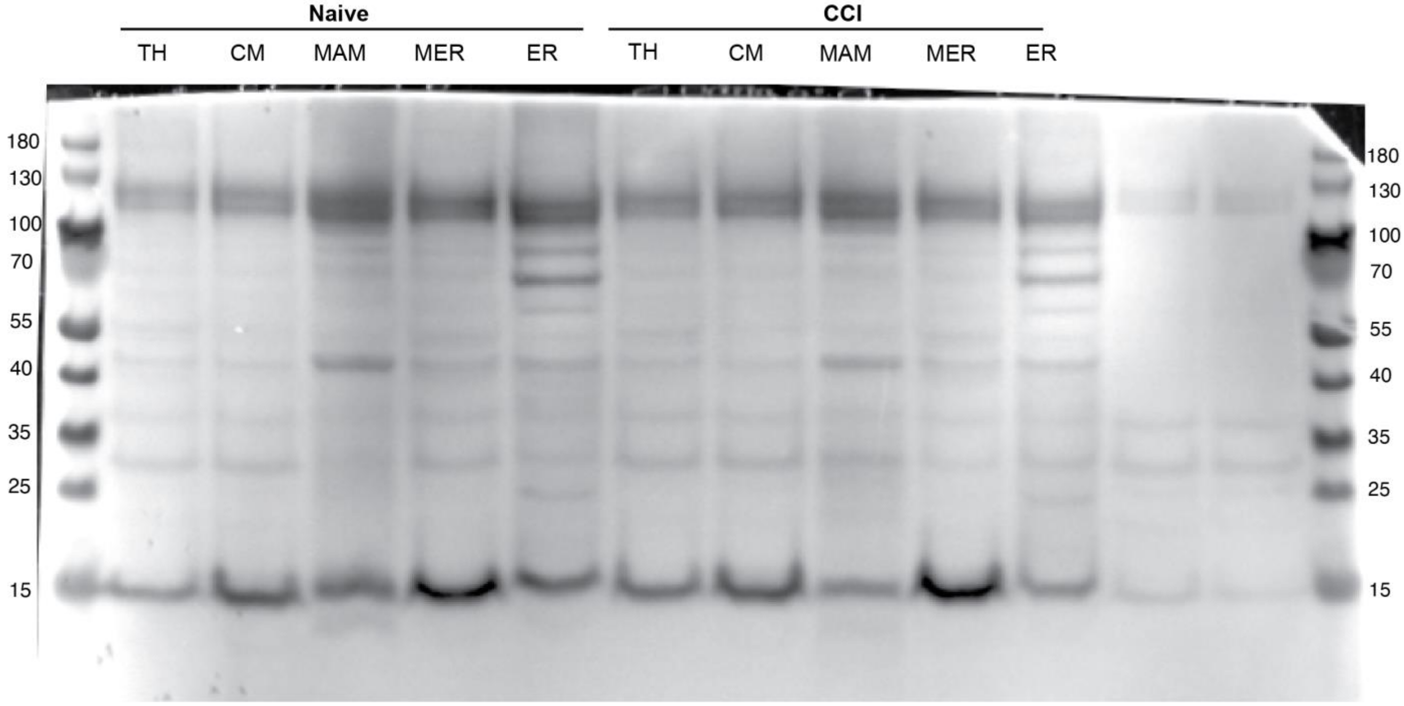

Fig. 2A: Western blot for APP-C99 (hippocampus)

M3.2 (Biolegend 805701) to detect APP-C99 (14 kDa)

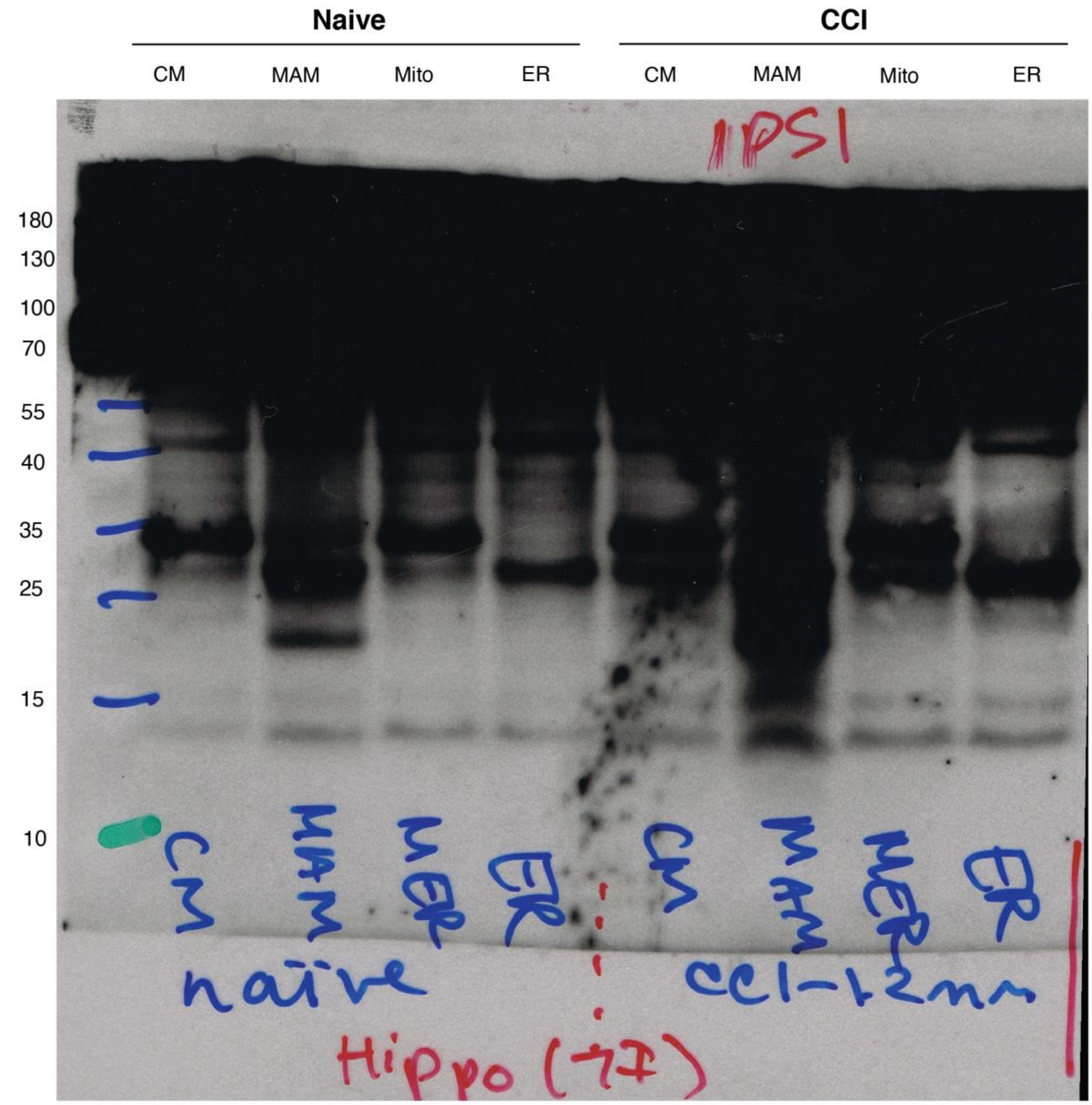

APP C-terminus (Sigma A8717) to detect APP-C99 (14 kDa) and APP-C83 (13 kDa)

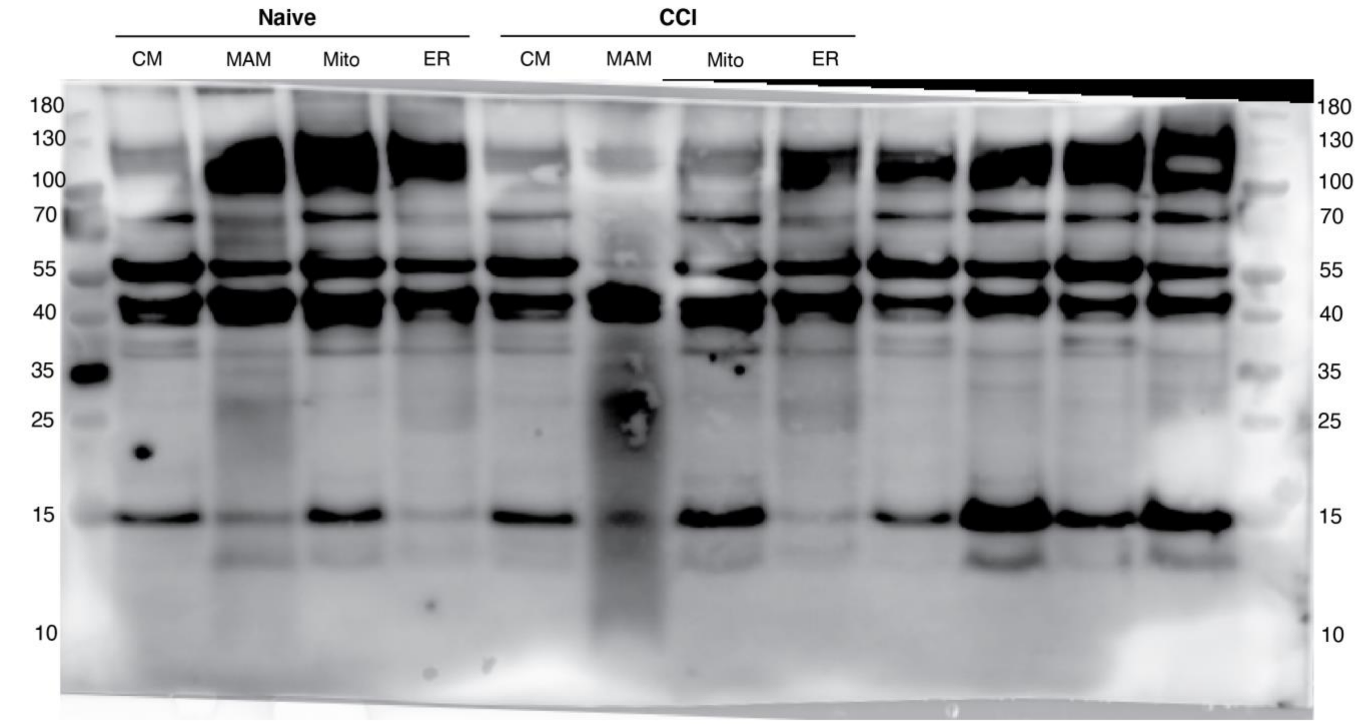

Erlin-2 (Abcam ab129207) - 43 kDa

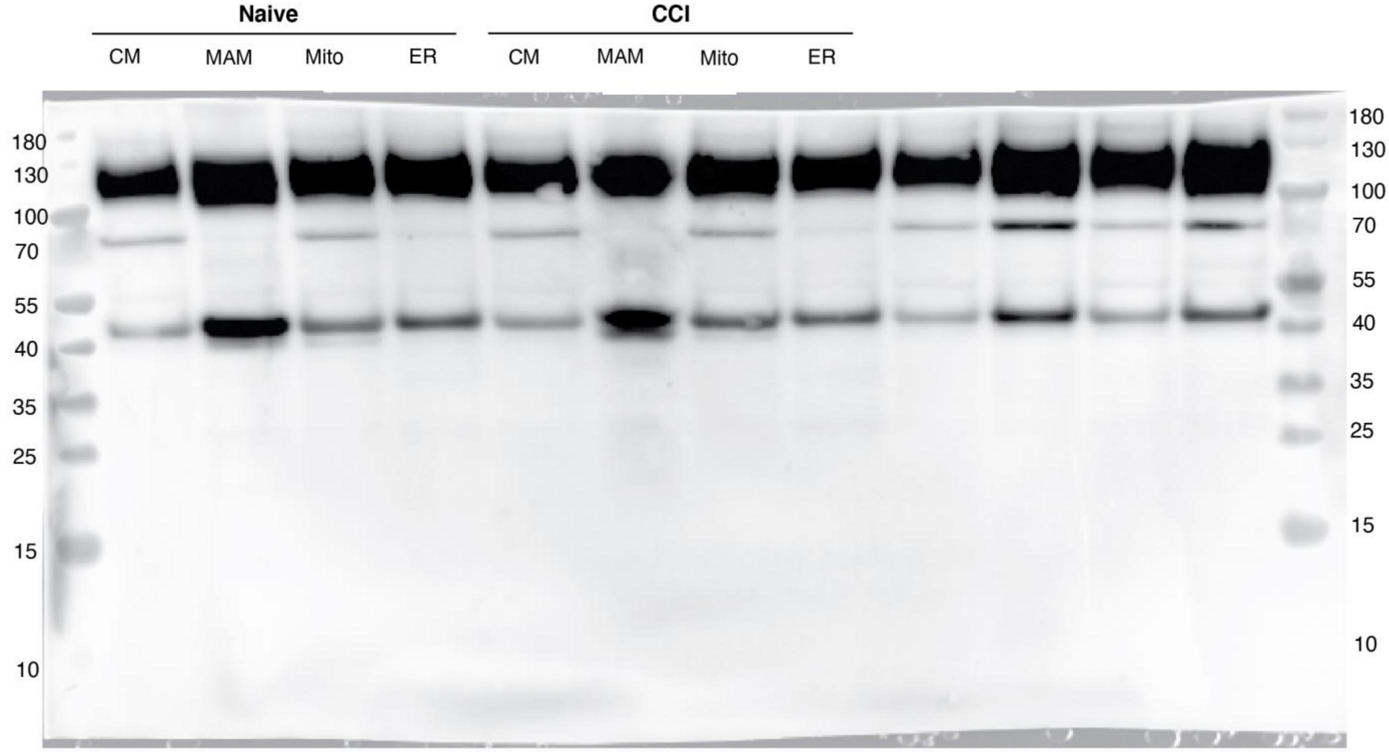

TOM20 (Santa Cruz sc-11415) - 16 kDa

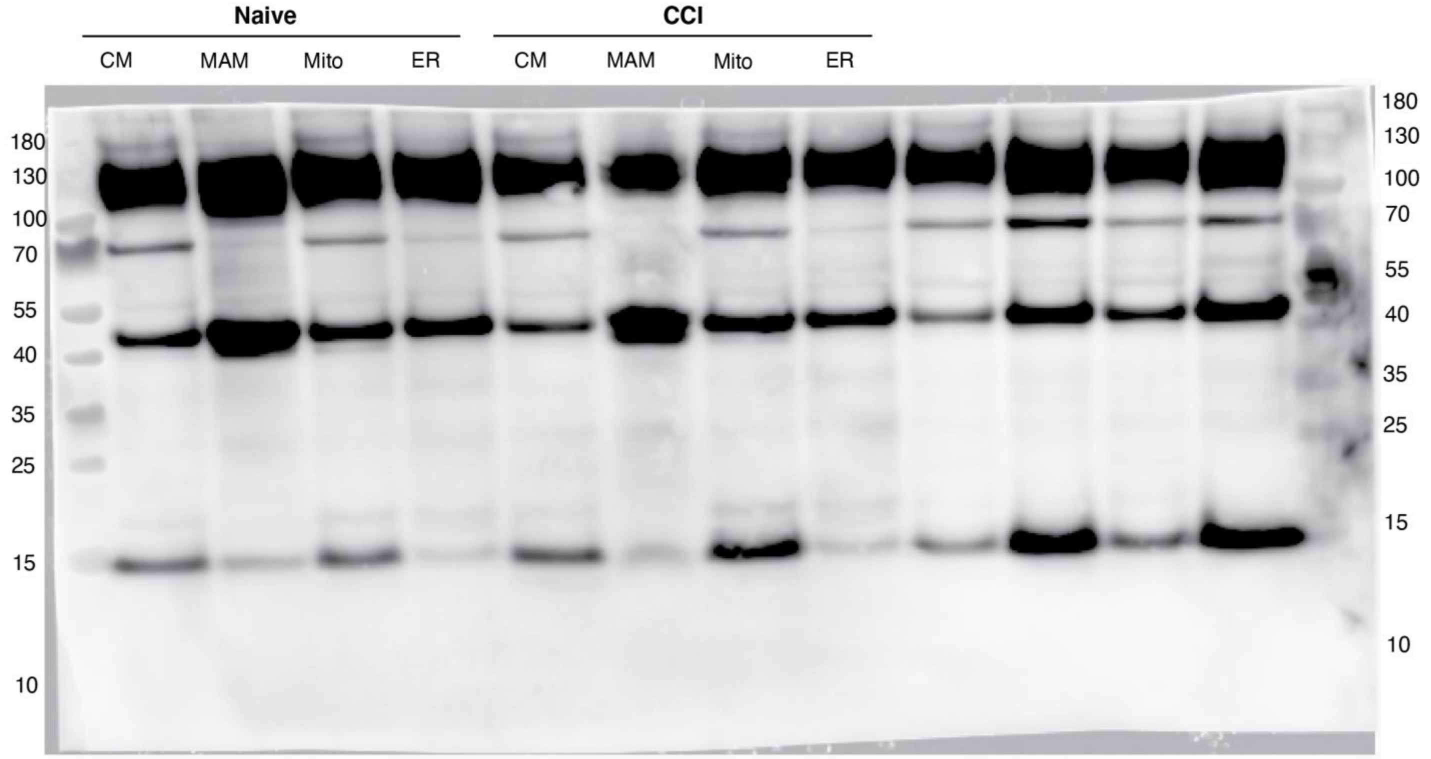

Fig. 5E and Supplemental Fig. 6F
Western blot for OxPhos complexes and TOM20

Cortex

Rodent OxPhos cocktail (Abcam ab110413)
- Complex I - 20 kDa
- Complex II - 30 kDa
- Complex III - 48 kDa
- Complex IV - 40 kDa
- Complex V - 55 kDa

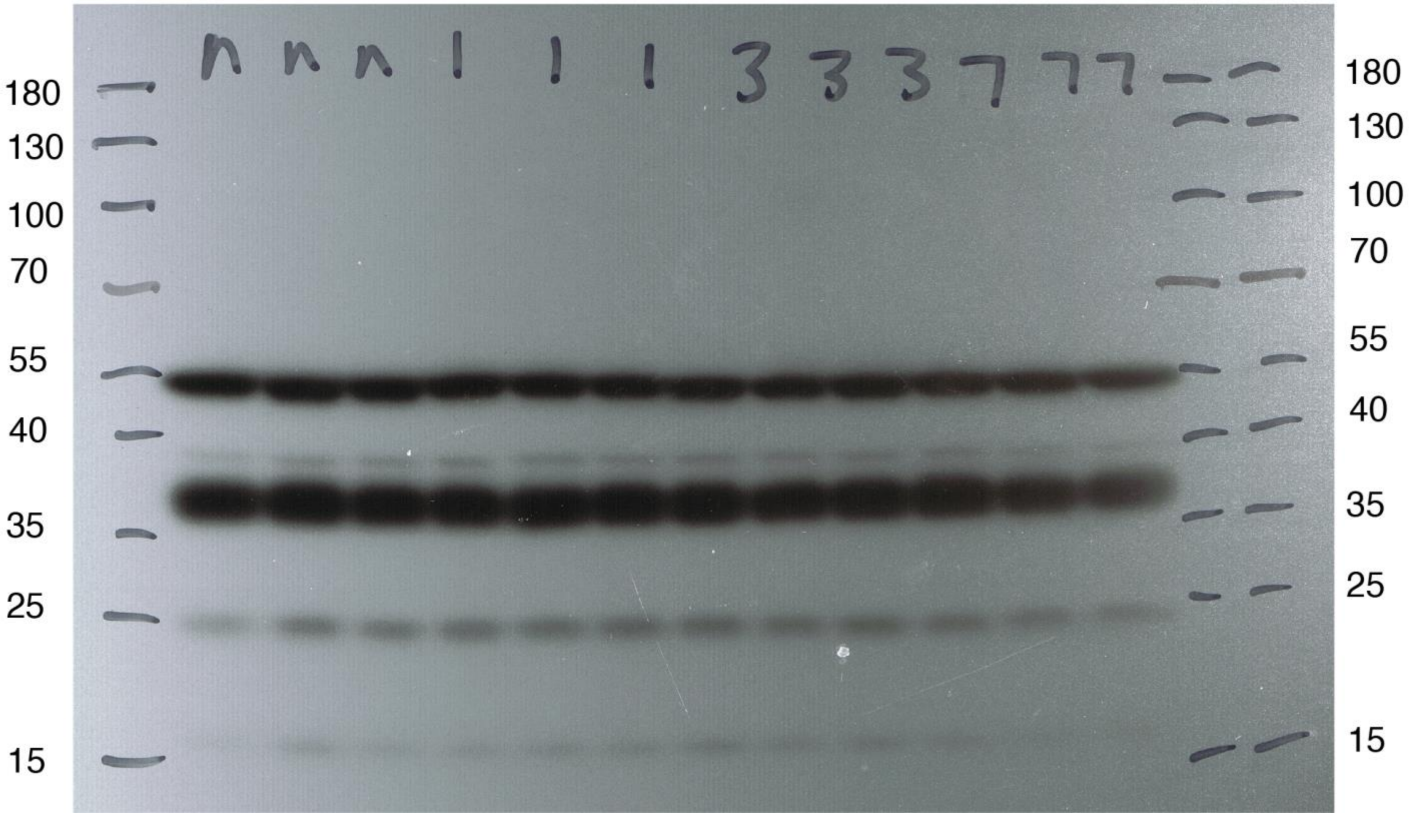

TOM20 (Santa Cruz sc-11415) - 16 kDa
B-actin (Sigma A5441) - 42 kDa

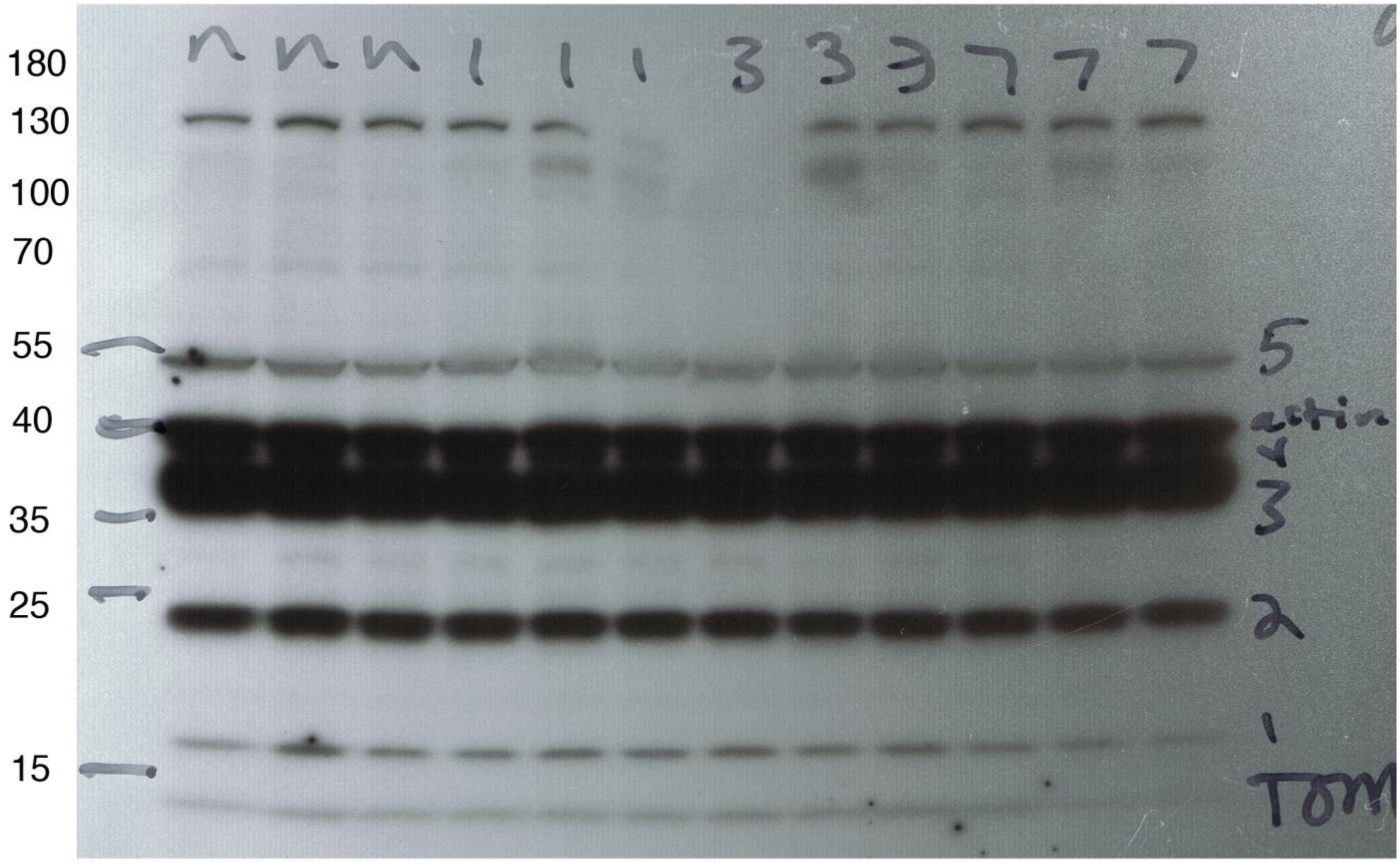

Fig. 5E and Supplemental Fig. 6F
Western blot for OxPhos complexes and TOM20

Hippocampus

Rodent OxPhos cocktail (Abcam ab110413)
- Complex I - 20 kDa
- Complex II - 30 kDa
- Complex III - 48 kDa
- Complex IV - 40 kDa
- Complex V - 55 kDa

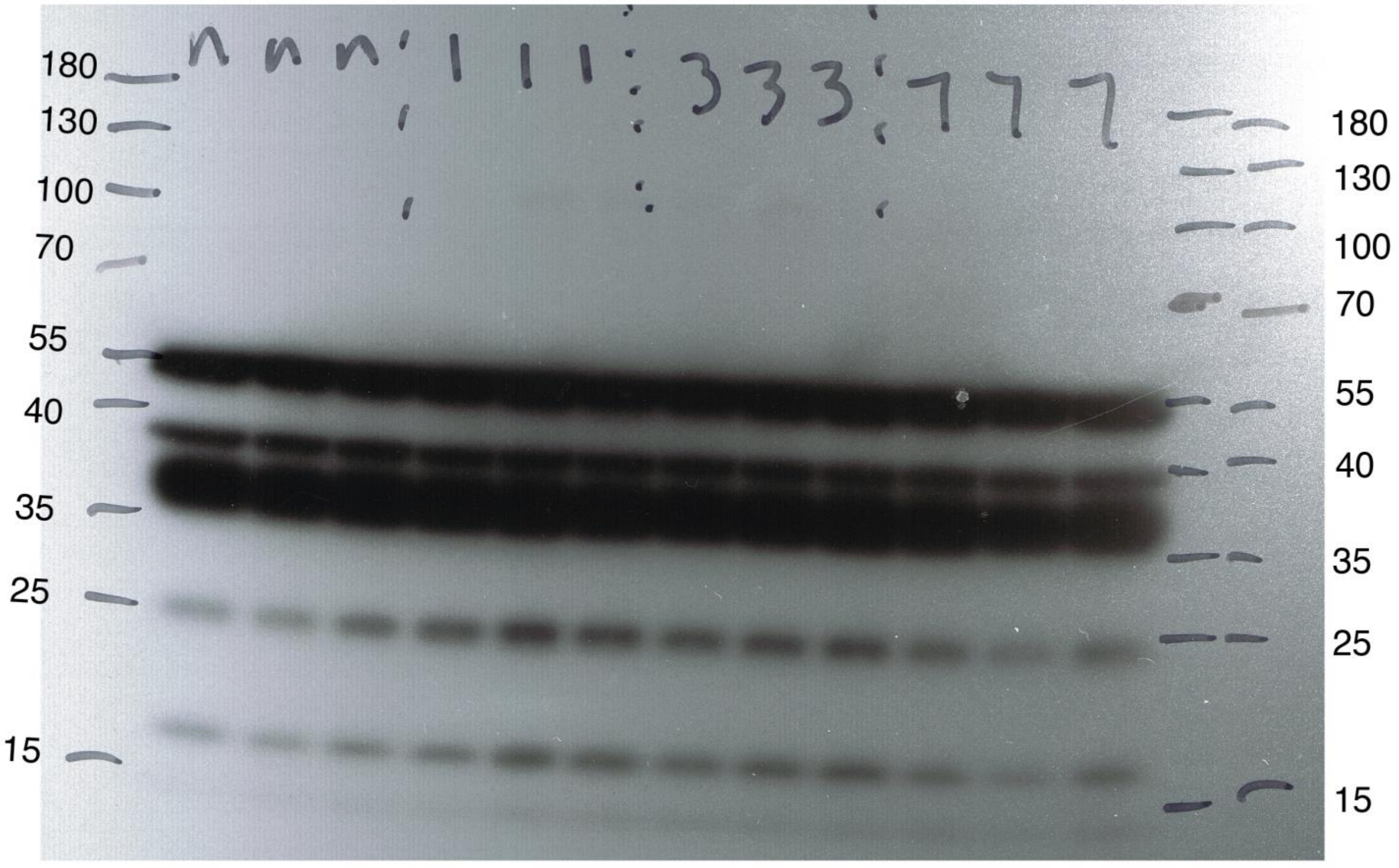

TOM20 (Santa Cruz sc-11415) - 16 kDa

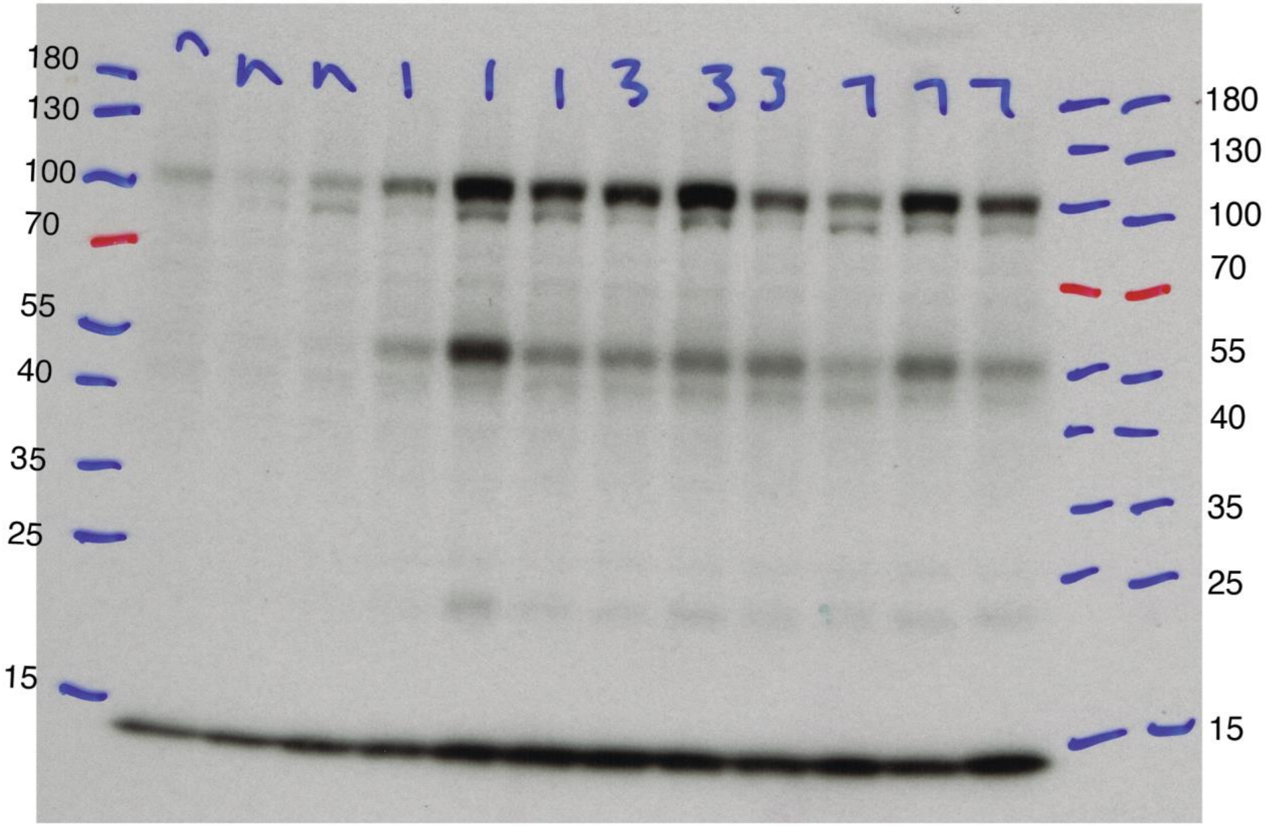

Vinculin (Sigma V4505) - 124 kDa

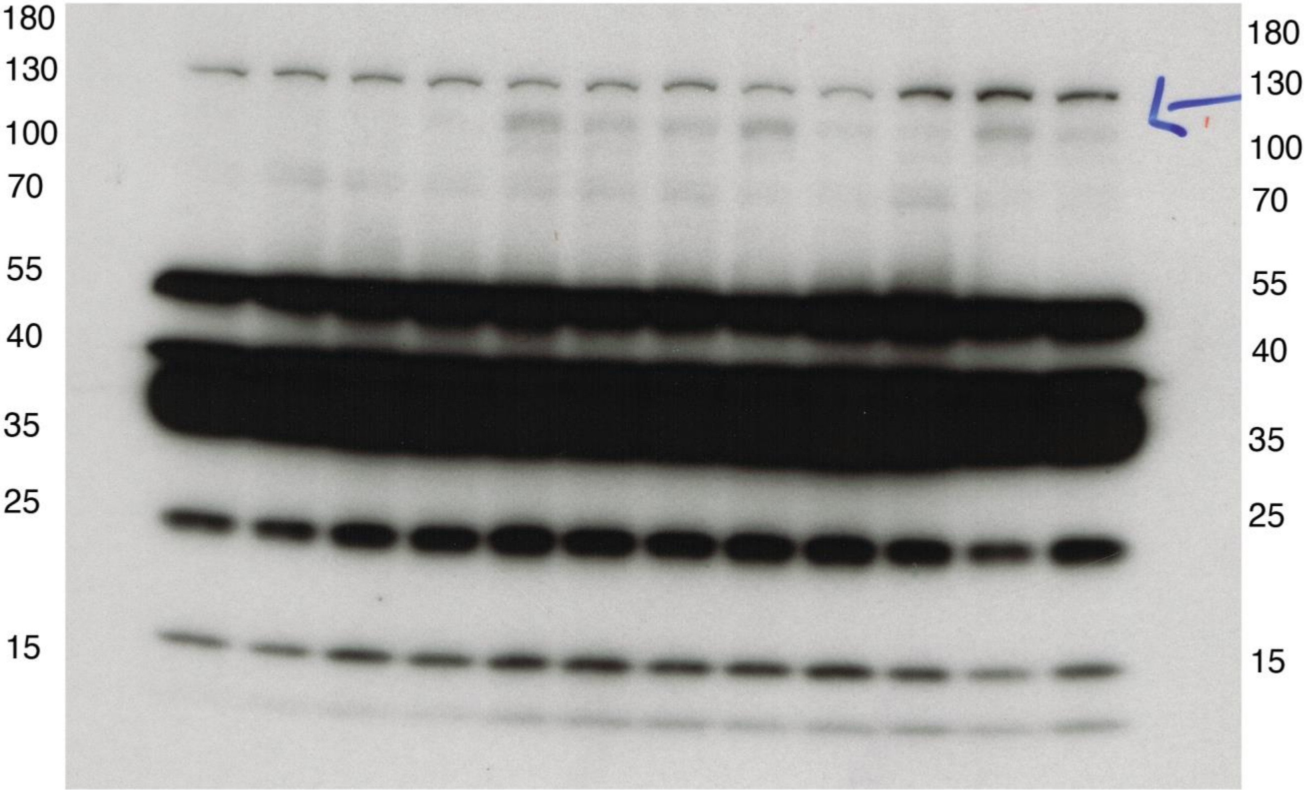

Supplement: Supplementary file 11 — Supplementary file11 (PDF 1579 KB) Original western blot images [file 10571_2022_1299_MOESM11_ESM.pdf]
